# A cerebellar-prepontine circuit for tonic immobility triggered by an inescapable threat

**DOI:** 10.1126/sciadv.abo0549

**Published:** 2022-09-28

**Authors:** Ashwin A. Bhandiwad, Nickolas C. Chu, Svetlana A. Semenova, George A. Holmes, Harold A. Burgess

**Affiliations:** Division of Developmental Biology, Eunice Kennedy Shriver National Institute of Child Health and Human Development, Bethesda, MD 20892, USA.

## Abstract

Sudden changes in the environment are frequently perceived as threats and provoke defensive behavioral states. One such state is tonic immobility, a conserved defensive strategy characterized by powerful suppression of movement and motor reflexes. Tonic immobility has been associated with multiple brainstem regions, but the underlying circuit is unknown. Here, we demonstrate that a strong vibratory stimulus evokes tonic immobility in larval zebrafish defined by suppressed locomotion and sensorimotor responses. Using a circuit-breaking screen and targeted neuron ablations, we show that cerebellar granule cells and a cluster of glutamatergic ventral prepontine neurons (vPPNs) that express key stress-associated neuropeptides are critical components of the circuit that suppresses movement. The complete sensorimotor circuit transmits information from sensory ganglia through the cerebellum to vPPNs to regulate reticulospinal premotor neurons. These results show that cerebellar regulation of a neuropeptide-rich prepontine structure governs a conserved and ancestral defensive behavior that is triggered by an inescapable threat.

## Teaser

When faced with imminent and inescapable danger, a conserved hindbrain neural circuit shuts down movement and sensory reflexes.

## INTRODUCTION

Sudden changes in the environment may signal new threats. In response, animals often avoid detection by predators and facilitate threat assessment by suppressing physical activity (*1*, *2*). Behavioral arrest is a common defensive strategy against predatory threat described in many vertebrate and invertebrate species (*3*–*5*) that forms part of the defense cascade, a continuum of behaviors that scale with perceived threat immediacy (*6*, *7*). Physical activity is suppressed at each end of the defense cascade as part of responses to both distant and immediate threat. When peril is relatively low, for example, at first detection of a predator cue, animals instigate orientation behaviors. In the post-encounter phase of defensive behaviors, animals exhibit “freezing” behavior, characterized by suppressed movement and heightened sensory acuity with increased readiness to flee. With escalating threat, animals initiate escape behaviors, and, lastly, when faced with imminent entrapment or actual capture, lapse into a catatonic-like state of “tonic immobility.” The defense cascade presents a valuable model for resolving neural mechanisms that select and coordinate the expression of competing behavioral programs.

Freezing and tonic immobility are distinct behaviors within the defense cascade. Both behaviors are defined by behavioral arrest but are triggered by different levels of perceived threat and have converse effects on sensory responsiveness, muscle tone, and postural control (*7*, *8*). The core circuit for freezing behavior consists of the periaqueductal gray, although additional regions including the parabrachial nuclei, cortex, and cerebellum have been implicated (*2*, *4*, *9*–*11*). In contrast, very little is known about the neural circuit basis for tonic immobility, a last-resort defensive response, induced by imminent threat or actual restraint, in which animals show diminished sensory and reflex responsiveness (*6*, *7*). Tonic immobility has been described in a wide variety of animal species under different names, including “phasic immobility,” “playing possum,” “death feigning,” and “thanatosis” (*12*). In humans, tonic immobility is linked to feelings of paralysis during traumatic events and is a major predictor of post-traumatic stress disorder severity (*13*). The duration of immobility varies widely between species, from as little as several seconds to more than an hour, and is sometimes interrupted by brief movement bouts (*14*). Paradoxically, animals in this state show increased EEG theta power, heart rate, and breathing rate, suggesting hyperarousal (*1*). Early decerebration studies showed that the circuit for restraint-induced tonic immobility was localized to the brainstem, but the specific neural substrates and circuit connectivity for inducing tonic immobility are still unknown (*15*). How extreme threats initiate tonic immobility, overriding the expression of alternative defensive behaviors, remains a fundamental question.

Both freezing behavior and tonic immobility occur in fishes. Freezing behavior has been studied as a response to electrical shock (*16*), exposure to alarm pheromones (*17*), and placement into a novel tank (*18*). Several studies have also reported a state of tonic immobility in fish that manifests as prolonged inactivity, loss of responsiveness, and suppression of righting reflexes, often after manual restraint or physical inversion (*14*, *19*). In larval stage zebrafish, immobility may be induced by an extreme vibratory stimulus well above intensity levels that normally induce escape responses, raising the possibility of applying circuit neuroscience methods to unraveling a neuronal substrate for this behavior (*20*).

Here, we demonstrate tonic immobility in larval zebrafish evoked by a persistent and inescapable threat and defined by behavioral arrest and reflex suppression. We performed a circuit-breaking screen (an unbiased neuronal ablation screen using transgenic Gal4 lines) to disrupt circuit function, followed by targeted ablations and imaging to map a contiguous sensorimotor pathway for this terminal defensive behavior from primary mechanosensory neurons via the cerebellum and vPPNs to premotor reticulospinal neurons. We show that vPPNs express multiple stress and homeostasis-related neuropeptides and are bilaterally activated by threatening stimuli. Last, we propose a circuit model where multimodal sensory inputs act through the cerebellum to bilaterally activate vPPNs and trigger behavioral arrest by disrupting RoL1 reticulospinal neuron activity. Together, these results reveal a previously unknown proposed sensorimotor circuit model for this highly conserved defensive behavior that is activated when the animal is faced with an inescapable threat.

## RESULTS

### Repeated vibratory stimuli evoke locomotor arrest

**Fig. 1.**
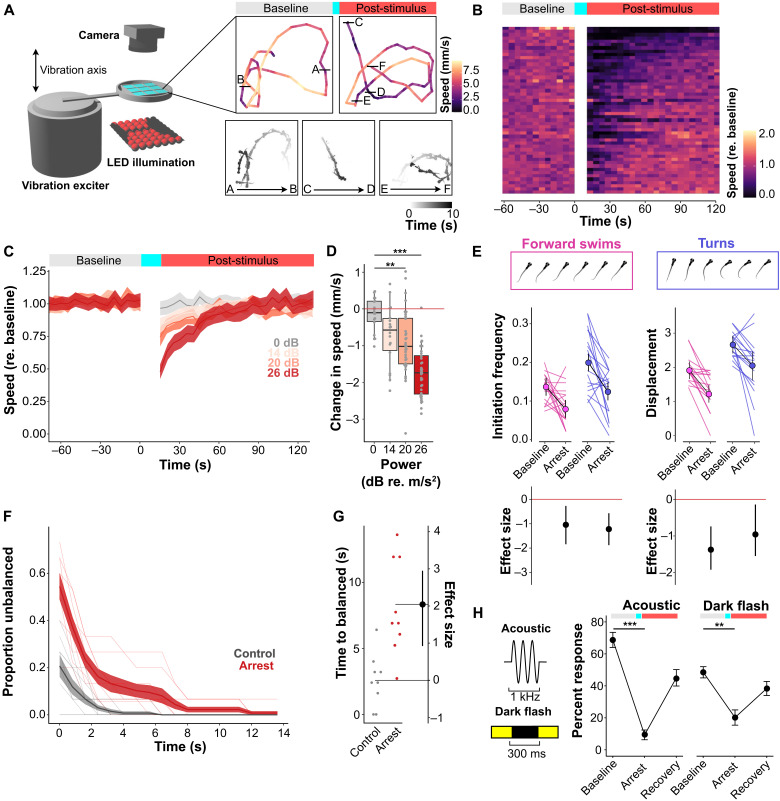
Repeated vibratory stimuli evoke tonic immobility in larval zebrafish. (**A**) Left: Schematic of the apparatus used to evoke behavioral arrest. Right, top: Locomotion traces from a single fish during baseline and post-stimulus phases. Color represents speed in each epoch. Right, bottom: Fish positions during 10-s epochs of baseline (A and B), arrest (C and D), and recovery (E and F) phases; saturation indicates time within epoch. (**B**) Raster plot of speed (mm/s; *n* = 51 fish). Rows: speed normalized to mean baseline. (**C**) Mean normalized speed after 15-s arrest-inducing stimuli of 0 dB (no stimulus), 14 dB, 20 dB, and 26 dB re. 1 m/s^2^ (*n* = 27 fish). Shaded area: SEM. (**D**) Speed change in first epoch (mm/s) compared to baseline at 0 dB (control), 14 dB, 20 dB, and 26 dB re. 1 m/s^2^ (*n* = 27 fish). Dunnett’s test **P < 0.01, ***P < 0.001. (**E**) Swim initiation frequency and displacement per swim for turns (blue) and forward swims (magenta). Top: Examples of turns and forward swims. Middle: Changes in initiation frequency (left) and displacement for each bout (right) during baseline and arrest (*n* = 16). Bottom: Effect sizes (Cohen’s *d*) for change in responsiveness, mean ± 95% confidence interval (CI). (**F**) Left: Righting reflex (*n* = 10 groups with five fish per group) under control (gray) and arrest (red) conditions. Shaded area: SEM. (**G**) Estimation plot for time to return to balance under control (gray) and arrest (red) conditions. (**H**) Left: Schematic of acoustic and dark flash stimuli. Right: Changes in acoustic short-latency startle (*n* = 27 fish, interstimulus interval = 30 s) and dark flash–evoked O-bend responses (*n* = 27 fish, interstimulus interval = 60 s) during arrest (means ± SEM). **P < 0.01, ***P < 0.001, Paired-sample *t* test.

Intense vibratory stimuli elicit a sustained decrease in locomotor activity in 6 days post-fertilization (dpf) zebrafish larvae (*20*). To characterize this state and determine its similarity to tonic immobility, we delivered repeated pulsed high-amplitude, low-frequency stimuli using a vibration exciter and measured changes in swim speed relative to a 60-s prestimulus baseline (Fig. 1, A and B). Individual pulses elicited escape responses with 96 ± 3% responsiveness and the stimulus was repeated 95 times over a 15-s period—this simulated a persistent and inescapable threat (movie S1). Following repeated stimulus presentation, larvae showed reduced swimming that scaled with stimulus amplitude (Fig. 1, C and D). This reduction recovered to the prestimulus baseline level over the subsequent minute, with a half-recovery time (T_0.5_) of 14 to 26 s (95% confidence interval, *n* = 27 larvae). For all subsequent experiments, we used a stimulus that decreased speed by 50% with a half-recovery time of 20 s among all animals tested (fig. S1, A to C). Behavioral arrest was defined as the change in speed between the prestimulus baseline average and the first epoch of the post-stimulus condition. This estimate is conservative because it samples a 5-s cumulative window and because noise in tracking tended to artificially inflate the amount of movement especially in stationary fish. High-speed video recordings showed that the 50% reduction in speed on the group level was driven by individual differences in sensitivity to the vibratory stimulus; some individuals showed prolonged immobility, others a reduction in speed, while a subset of larvae showed no response (fig. S1B). Although recovery appeared gradual when mean speed was averaged across larvae, recovery was abrupt in individual animals, with variable latencies for the transition from immobility to resumption of normal swimming (fig. S1, C and D).

After the intense stimulus, the remaining swim activity showed relatively straight trajectories compared to the looping paths observed during baseline conditions (Fig. 1A and fig. S1E). Kinematic analysis of high-speed video indicated that this was due to a change in swim bout type usage. Spontaneous movement in larvae primarily occurs as discrete bouts of slow forward swims and routine turns. Consistent with the switch to straight swim trajectories, during arrest, larvae showed a large reduction in the frequency of turn and forward swim initiations (Fig. 1E). Displacement per movement was also decreased (Fig. 1E). Thus, during vibration-induced arrest, the decreased movement is due to a reduction in movement initiation and in the travel distance of each event.

In other species, reduced movement in response to threat may be classified as freezing or tonic immobility on the basis of differential effects on sensorimotor reflexes including modulation of the righting reflex, a vestibular reflex that restores dorsoventral orientation when the animal is restrained and inverted (*21*). In general, freezing facilitates, whereas tonic immobility, suppresses sensorimotor responses (*22*, *23*). Therefore, to differentiate between freezing and tonic immobility, we investigated changes to the righting reflex during behavioral arrest.

At baseline, zebrafish consistently exhibit a dorsal-up posture. As in other animals, inversion or forced side-lying evokes a vestibulomotor self-righting reflex to restore the dorsal-up posture (*24*). We tested whether the righting reflex was suppressed during behavioral arrest by presenting a brief high-intensity low-frequency stimulus that disrupted balance. Destabilizing larvae during the arrest state increased the likelihood of inversion and delayed restoration of the dorsal-up posture compared to baseline, demonstrating a disruption of the righting reflex, consistent with tonic immobility (Fig. 1, F and G). We analyzed changes in acoustic-evoked startle and visual reflexes after the arrest-evoking stimulus in Fig. 1B and observed that both acoustic and visual reflexes showed significantly reduced responsiveness during the arrest period (Fig. 1H). Thus, after intense vibration, zebrafish show arrested movement, suppression of sensorimotor responses, and loss of the righting reflex, all characteristic features of tonic immobility as manifest in other species under extreme threat.

Early work in rabbits suggested that immobility may reflect inhibition of spinal motor circuits (*15*). We tested whether motor circuit function was altered during arrest by direct activation of Mauthner cells, which drive short-latency startle responses, with an electrical pulse stimulus (*25*). We reasoned that if spinal motor circuits were inhibited during tonic immobility, Mauthner cell–mediated startle response kinematics would be disrupted, leading to lower amplitude movements. Electric pulse-initiated escapes were normal (fig. S1F) and kinematic analysis of remaining visual and acoustic responses performed during arrest showed no changes in escape bend angle or displacement (fig. S2). It is therefore unlikely that motor circuits are directly suppressed during behavioral arrest. Rather, reduced movement reflects a change in premotor activity that initiates and sustains movement.

**Fig. 2.**
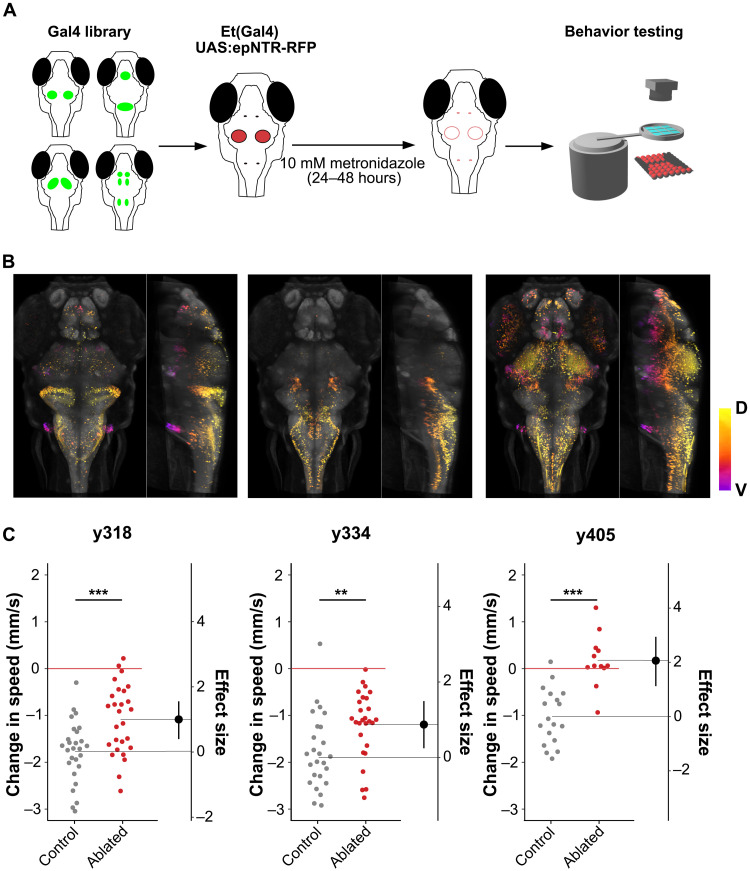
A circuit-breaking screen for behavioral arrest. (**A**) Schematic for the circuit-breaking screen using 31 enhancer-trap Gal4 lines. (**B**) Dorsal and sagittal maximum projection of Gal4 patterns in three lines, *y318-Gal4* (left), *y334-Gal4* (middle), and *y405-Gal4* (right), which showed disruption of arrest following stimulation. Projections are depth-coded, D, dorsal; V, ventral. Gray counterlabel: *HuC:Cerulean*. (**C**) Change in locomotion after intense vibration for lines shown in (B) for ablated larvae (red) and intact sibling controls (gray). *y318-Gal4* (*n* = 23 control/26 ablated), *y334-Gal4* (*n* = 21/24), *y405-Gal4* (*n* = 18/12). Effect sizes are Cohen’s *d*. **P < 0.01, ***P < 0.001, independent samples *t* test.

To reveal neurons that mediate behavioral arrest, we conducted a circuit-breaking screen using a library of transgenic Gal4 enhancer trap lines (*26*). Fish expressing Gal4 in specific neuronal populations were crossed to transgenic *UAS:epNTR-RFP*, a variant of nitroreductase that converts the prodrug metronidazole into a cell-specific toxin (Fig. 2A) (*27*). We screened 31 Gal4 lines and recovered three lines (*y318-Gal4*, *y334-Gal4*, and *y405-Gal4*) where vibration-induced arrest was diminished in ablated larvae [Fig. 2, B and C, and fig. S3A; full three-dimensional (3D) expression can be visualized at zbbrowser.com]. Ablations did not affect baseline activity in *y318-Gal4* or *y334-Gal4*, whereas spontaneous swimming was reduced in *y405-Gal4* (fig. S3B). Note that reduced spontaneous swimming in *y405-Gal4*–ablated larvae may contribute to an overestimate of the effect of ablation in this line. All three lines showed a similar recovery time to controls (fig. S3C), suggesting that the underlying neurons initiate arrest rather than regulate the duration of immobility. Furthermore, disruption of vibration-induced arrest did not generalize to electric shock–induced freezing, indicating that vibration-induced arrest was independent of a previously described pathway for freezing behavior (fig. S4) (*16*).

### Mechanosensory inputs induce arrest via a cerebellar pathway

**Fig. 3.**
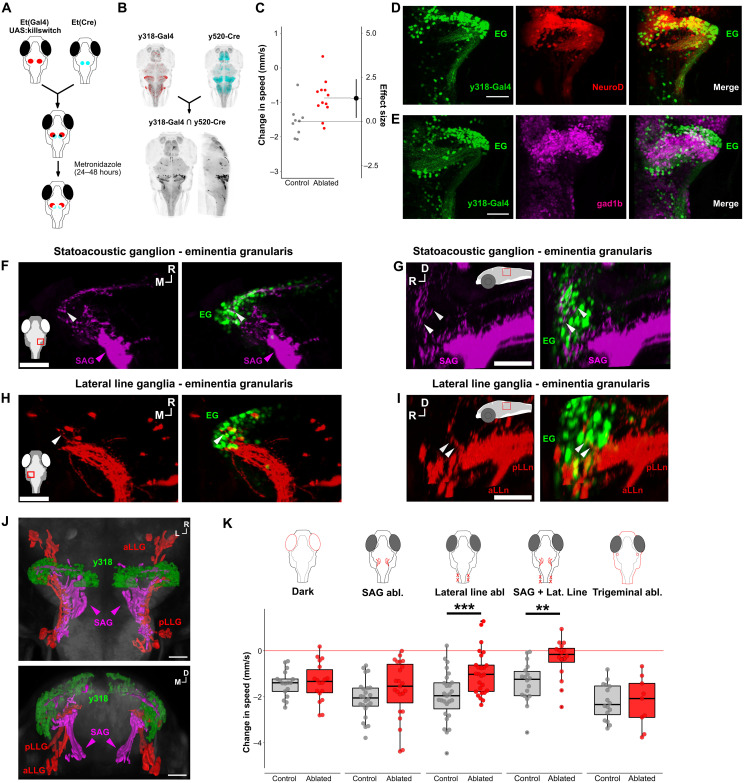
Acoustic and lateral line inputs converge onto lateral cerebellar granule cells to drive arrest. (**A**) Schematic of intersectional ablation strategy to parse *y318-Gal4* expression. (**B**) Maximum projections of *y318-Gal4* (red) and *y520-Cre* (cyan) expression patterns. Bottom: Dorsal and sagittal views of neurons labeled by NTR in *y318-Gal4*/*y520-Cre* intersect (mean from *n* = 3 fish). (**C**) Swim speed change (mm/s) after stimulus in *y318-Gal4*/*y520-Cre*–ablated fish (red, *n* = 12) and sibling controls (gray, *n* = 9). Effect size is Cohen’s *d*. *t* test *P* = 0.01. (**D**) Confocal slice of cerebellum showing *y318-Gal4*;*UAS:kaede* (green) and *NeuroD-eGFP* (red), which labels cerebellar granule cells. Scale bar, 50 μm. (**E**) Confocal slice of cerebellum showing *y318-Gal4*;*UAS:kaede* (green) and *gad1b:RFP* (magenta; GABAergic neurons). Scale bar, 50 μm. (**F** and **G**) Confocal slice of *y256-Gal4*;*UAS:eGFP-CAAX*–labeled neurons (magenta) from SAG (magenta arrowhead) coregistered with *y318-Gal4;UAS:kaede* (green). White arrowhead: neurite termination of *y256-Gal4* neurons. Inset: imaged area. Scale bar, 30 μm. (**H** and **I**) Confocal slice of *y397-Gal4*;*UAS:eGFP-CAAX*–labeled neurons from the anterior and posterior lateral line ganglia (red) coregistered with *y318-Gal4;UAS:kaede* (green). White arrow: *y397-Gal4* neuron termination. Scale bar, 30 μm. (**J**) 3D reconstruction of SAG neurons from (F) and (G) (magenta), aLLG and pLLG neurons (red) from (H) and (I), and *y318-Gal4*;*UAS:kaede* (green). Scale bar, 50 μm. (**K**) Effects of removing visual (dark; *n* = 17 control, *n* = 18 dark), auditory (SAG Abl; *n* = 23 control, *n* = 24 ablated), lateral line (lateral line abl; *n* = 27 control, *n* = 26 ablated), combined SAG and lateral line (*n* = 18 control, *n* = 16 ablated), and somatosensory cues (Trigeminal abl; *n* = 16 control, *n* = 9 ablated) on arrest. *t* test, **P < 0.01, ***P < 0.001. Abbreviations: aLLn, anterior lateral line; EG, eminentia granularis; pLLn, posterior lateral line; SAG, statoacoustic ganglion; D, dorsal; R, rostral; M, medial; L, lateral.

Each of the three Gal4 lines labeled neurons in multiple brain regions. To isolate the arrest-related neurons within these lines, we first analyzed *y318-Gal4*, which had prominent expression in the cerebellum accompanied by sparse clusters in other brain regions. We used an intersectional method to selectively label and ablate the cerebellar cluster by crossing *y318-Gal4*, *UAS:lox-GFP-lox-epNTR-RFP* fish to *y520-Cre* that has prominent overlapping expression only in the cerebellum (*28*). Ablation of cerebellar neurons in *y318-Gal4* (Fig. 3, A and B) significantly reduced behavioral arrest following vibratory stimuli (Fig. 3C) with a similar effect size to complete *y318-Gal4* ablation, indicating that the cerebellar neurons are the relevant subpopulation. *y318-Gal4* cerebellar neurons colocalized with green fluorescent protein (GFP) in *NeuroD:eGFP* transgenic larvae, a known marker of granule cells (Fig. 3D) (*29*), and were largely distinct from *gad1b*-expressing cerebellar neurons (Fig. 3E). We note that *y318-Gal4* ablation did not show a complete loss of function because of the mosaic labeling of cells within the structure (Fig. 3D). These data identify *y318-Gal4* cerebellar granule cells as a component of the arrest-induction circuit.

Involvement of the cerebellum in initiating arrest led us to investigate input and output pathways. Granule cells receive direct input from auditory/vestibular and lateral line ganglia in multiple fish species (*30*). Given that vibrational stimuli were used to evoke arrest, we asked whether primary sensory afferents also project to the cerebellum in zebrafish. We labeled central projections from the lateral line ganglia and the statoacoustic ganglion by expressing membrane-tagged GFP in *y397-Gal4, UAS:eGFP-CAAX* larvae, and *y256-Gal4, UAS:eGFP-CAAX* larvae, respectively. As in other fish species, auditory (Fig. 3, F and G) and lateral line (Fig. 3, H and I) afferents both terminated near the granule cells of the eminentia granularis (Fig. 3J).

Input from the auditory and lateral line systems into the cerebellum suggested that mechanosensory inputs are important for evoking arrest. As our arrest-inducing stimulus potentially activated multiple sensory modalities, we tested the role of visual, acoustic/vestibular, lateral line, and somatosensory systems. We disrupted auditory and vestibular inner ear function by ablating posterior statoacoustic ganglion neurons labeled by *y256-Gal4*, *UAS:epNTR-RFP* and found no effect on arrest (Fig. 3K and fig. S5A). We then ablated the flow-sensing lateral line neuromasts using bath application of 250 μM neomycin (fig. S5B). Neomycin ablation reduced arrest by 30% compared to nontreated controls with no changes in baseline movement. However, combined ablation of posterior auditory afferents and lateral line neuromasts led to a total loss of arrest, indicating that both flow and acoustic/vestibular information coordinately drive arrest. In contrast, arrest was induced normally when we conducted the experiment in darkness to remove visual cues or disrupted somatosensory inputs by ablating trigeminal ganglion neurons. These data indicate that inner ear and lateral line signals evoke behavioral arrest in this context via inputs to eminentia granularis granule cells, a well-described pathway in fishes (*31*).

### Glutamatergic prepontine neurons are critical for arrest

**Fig. 4.**
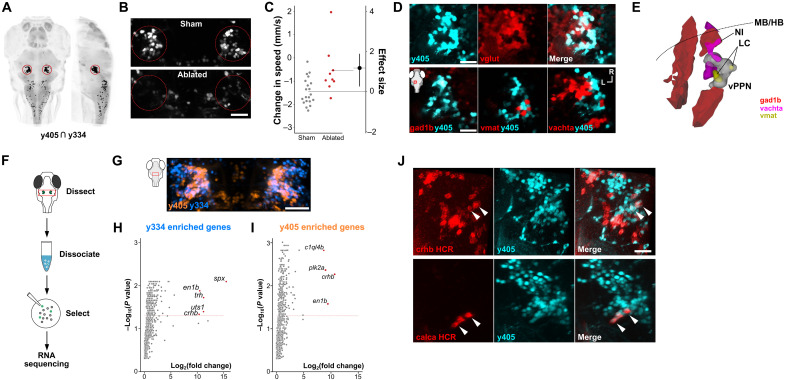
Neuropeptide-rich neurons in the prepontine tegmentum are required for behavioral arrest. (**A**) Dorsal and sagittal views of computationally predicted overlap between *y334-Gal4* and *y405-Gal4* (black pixels). vPPNs are outlined. *HuC:Cerulean* counterlabel in gray. (**B**) Confocal images from *y405-Gal4* after sham ablation or bilateral vPPN multiphoton ablation. Scale bar, 40 μm. (**C**) Swim speed change after intense vibration in control (gray, *n* = 20) and *y405-Gal4* vPPN–ablated (red, *n* = 10) larvae. Effect size is Cohen’s *d*. *t* test, *P* < 0.001. (**D**) Top: Confocal section of *y405-Gal4*;UAS:kaede (cyan) and *vglut:GFP* (glutamatergic neurons, red). Scale bar, 25 μm. Bottom: Overlay of *y405-Gal4* (cyan) with *gad1b:RFP* (left), *vmat2:GFP* (middle), and *vachta:GFP* (right) neurons. Inset: imaging window. Scale bar, 25 μm. (**E**) Schematic 3D reconstruction of cell clusters in the prepontine tegmentum based on imaging *y405-Gal4* vPPNs (white), noradrenergic locus coeruleus neurons (LC, *vmat2:GFP*, yellow), cholinergic neurons of the nucleus of the isthmus (NI, *vachta:GFP*, magenta), and GABAergic neurons (*gad1b:RFP*, red). Midbrain-hindbrain boundary (MB/HB, dotted line). (**F**) Procedure for manual isolation of vPPNs for transcriptomic analysis. (**G**) Maximum projection of *y334-Gal4* (blue) and *y405-Gal4* (orange) expression in vPPNs. Scale bar, 40 μm. (**H** and **I**) Enrichment of genes expressed in *y334-Gal4* (H) and *y405-Gal4* (I) relative to a pan-neuronal reference. Labeled genes (red) show log_2_ fold changes >8 and false discovery rate–corrected *P* values < 0.05. Dashed red line: Benjamini-Hochberg corrected 5% false discovery rate. (**J**) Top: *crhb* mRNA (red) labeled using hybridization chain reaction (HCR) with *y405-Gal4;UAS:kaede* (cyan). Arrowheads show example neurons that are colabeled by kaede and *crhb*. Scale bar, 30 μm. Bottom: calca mRNA (red) labeled using HCR with *y405-Gal4;UAS:kaede* (cyan). Arrowheads show example neurons that are colabeled by kaede and calca. Scale bar, 30 μm.

Having established that inner ear and lateral line signaling to cerebellar granule cells form part of the circuit that initiates arrest, we searched for other components of the circuit by examining arrest-related neurons within *y405-Gal4* and *y334-Gal4*, the two other lines recovered in our screen. Although these lines express Gal4 in multiple brain regions, a cluster of ventral prepontine neurons (vPPNs) were labeled in both, making them strong candidates (Fig. 4A and fig. S6). Targeted multiphoton laser ablation of *y334-Gal4* vPPNs significantly decreased arrest (Fig. 4, B and C) with a similar magnitude to *y334-Gal4* ablation (Fig. 2C).

vPPNs are located in an anterior part of the hindbrain that comprises multiple neuronal cell groups, including glutamatergic, GABAergic, and cholinergic neurons and the noradrenergic locus coeruleus. vPPNs labeled by *y334-Gal4* and *y405-Gal4* showed considerable overlap, labeling a continuous crescent-shaped arc within the prepontine hindbrain, with *y334-Gal4* labeling more neurons in the rostral and lateral part of the crescent (Fig. 4G). To further characterize vPPNs, we performed colocalization imaging experiments with transgenic lines that label major neurotransmitters (Fig. 4D). The majority of *y405-Gal4* vPPNs (97%, 288 of 296 cells from *n* = 5 fish) colocalized with *vglut2a:GFP*, indicating that these neurons are primarily glutamatergic. vPPNs did not colocalize with GABAergic neurons labeled by *gad1b:RFP* and were adjacent to, but did not overlap with cholinergic neurons of the nucleus isthmi labeled by *vachta:GFP* (*32*). Cells expressing *vmat2:GFP*, which labels the locus coeruleus, were interspersed with caudal vPPNs, but we did not find vPPNs that coexpressed GFP (*n* = 108 neurons; Fig. 4, D and E). To probe vPPN identity, we analyzed transcriptomes of neurons manually isolated from the prepontine region in *y334-Gal4* and *y405-Gal4* larvae using RNA sequencing (Fig. 4, F and H and I). Confirming that we correctly isolated prepontine neurons from these lines, both *y334-Gal4* and *y405-Gal4* transcriptomes were enriched in expression of *engrailed 1b* (*en1b*), an established midbrain-hindbrain boundary marker (*33*). Among the most highly enriched transcripts in vPPNs were several stress-associated neuropeptides, including corticotropin-releasing hormone b (*crhb*), spexin (*spx*), thyrotropin-releasing hormone (*trh*), and urotensin 1 (*uts1*; Fig. 4, H and I). We confirmed that *crhb* is expressed in *y405-Gal4* vPPNs using fluorescence in situ hybridization; 24% of vPPNs (274 of 1124 *y405-Gal4* neurons from *n* = 6 fish) coexpressed *crhb* transcripts, primarily in caudal vPPNs (Fig. 4J). Recent studies have implicated prepontine calcitonin gene-related peptide (CGRP)-expressing neurons in threat-related behaviors (*34*). We observed sparse expression of *calca*, the gene encoding CGRP, in both *y405-Gal4* and *y334-Gal4*; subsequent hybridization chain reaction (HCR) in situ mRNA labeling revealed that *calca* is expressed in a subpopulation of caudal vPPNs (*n* = 49 of 916 cells from *n* = 6 fish; Fig. 4J). Together, these data define a previously unknown area for defensive responses in the prepontine tegmentum, bounded caudally by the locus coeruleus, laterally by the nucleus isthmi, and consisting of glutamatergic cells that coexpress multiple neuropeptides.

### GABAergic inhibition of prepontine neurons is required for arrest onset

**Fig. 5.**
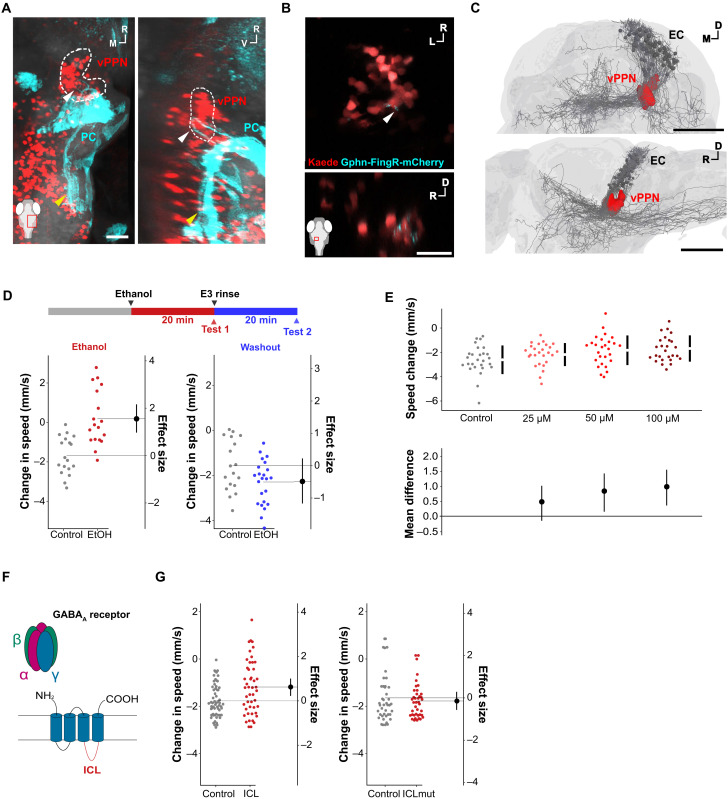
GABAergic signaling to vPPNs regulates arrest onset. (**A**) Dorsal (left) and sagittal (right) views of coregistered images of Purkinje cells (PC, *aldoca:Gal4*;*UAS:eGFP-CAAX*, cyan) and *y334-Gal4* vPPNs (dashed white line). Projections from PCs toward vPPNs (white arrowheads) and to hindbrain (yellow arrowheads). Scale bar, 50 μm. (**B**) Dorsal (top) and sagittal (bottom) confocal slices from *y405-Gal4*, *UAS:kaede* (red), and *UAS:gphn-FingR-mCherry* (cyan) transgenic larvae. Arrowheads show postsynaptic gephyrin puncta. Inset shows imaging window. Scale bar, 30 μm. (**C**) Dorsal (top) and sagittal (bottom) views of 3D reconstructions of eurydendroid cell projections (EC; gray) coregistered with *y334-Gal4* vPPNs (red). Scale bar, 100 μm. (**D**) Top: Schematic diagram of ethanol exposure experiment. Bottom, left: Change in swim speed (mm/s) after vibratory stimulus compared to average baseline in control (gray, *n* = 18) and 300 mM ethanol exposed (red, *n* = 18) shown as change in speed following vibrational stimuli. Effect size is Cohen’s *d*. *t* test, *P* < 0.001. Bottom, right: Change in swim speed (mm/s) in control (gray, *n* = 18) and ethanol washout (blue, *n* = 18) fish. Effect size is Cohen’s *d*. *t* test, *P* = 0.72. (**E**) Change in swim speed (mm/s) after bath application of GABA (*n* = 27 larvae per group). Top row shows raw change in speed and bottom row shows 95% CI of mean differences relative to control. ANOVA, *P* = 0.01. (**F**) GABA_A_ receptor complex and ICL of γ subunit. (**G**) Left: Changes in swim speed (mm/s) following vibratory stimuli in *y334-Gal4;UAS:ICL-GFP* (ICL, red, *n* = 47) and sibling controls (gray, *n* = 61). *t* test, *P* = 0.007. Right: Changes in swim speed (mm/s) following vibratory stimuli in *y334-Gal4;UAS:ICLmut-GFP* (ICLmut, red, *n* = 40) and sibling controls (gray, *n* = 47). *t* test, *P* = 0.91.

vPPNs are located in the tegmentum directly below the cerebellum, raising the possibility of a direct connection as part of the arrest circuit. We therefore examined efferent projections of cerebellar Purkinje cells in *aldoca:Gal4, UAS:eGFP-CAAX* transgenic larvae. Along with the previously reported caudal projection to the octaval nuclei (Fig. 5A, yellow arrowheads) (*35*), we noticed a small rostroventral projection from lateral Purkinje neurons. These Purkinje cell projections terminated adjacent to caudal vPPNs (Fig. 5A, white arrowheads) and suggested that Purkinje cells are presynaptic partners of vPPNs. We note that the *aldoca:Gal4* line used in these experiments mosaically labels Purkinje cells, and the Purkinje cell projections to vPPNs are incomplete. Because Purkinje cells are GABAergic, we asked whether vPPNs receive inhibitory inputs. Labeling postsynaptic inhibitory synapses formed onto vPPNs in *y334-Gal4*, *UAS:gephyrin-FingR-mCherry* transgenic larvae (*36*) revealed mCherry fluorescence in a subset of vPPNs, in a caudal region similar to the area receiving Purkinje neuron projections (Fig. 5B, white arrowheads), supporting the idea that these neurons receive inhibitory input. To find other possible connections between the cerebellum and vPPNs, we looked for possible projections to vPPNs from glutamatergic eurydendroid cells, which are analogous to the deep cerebellar nuclei in mammals and receive inhibitory inputs from Purkinje cells (*35*, *37*). We coregistered a single-neuron dataset containing eurydendroid cell projections (*38*); eurydendroid cell neurites passed through the vPPN region, suggesting a possible excitatory link between the cerebellum and prepontine neurons (Fig. 5C).

Because vPPNs showed evidence of gephyrin puncta, we pharmacologically manipulated GABAergic signaling to test whether GABA is required for arrest circuit function. Treatment with either GABA or ethanol, an indirect GABA_A_ receptor agonist, reduced vibration-induced behavioral arrest (Fig. 5, D and E). After ethanol washout, larvae recovered fully within 20 min. Because bath applied treatments likely affected GABA signaling throughout the brain and because ethanol can affect multiple non-GABAergic signaling pathways, we tested whether suppressing GABA_A_ receptor signaling specifically in vPPNs suppressed arrest. To do so, we adapted a strategy previously described in *Xenopus* in which transgenic expression of the intracellular loop (ICL) from the GABA_A_ receptor γ2 subunit reduces (but may not eliminate) GABA_A_ receptor–elicited postsynaptic currents (*39*). We generated a *UAS:ICL-GFP* construct to express the γ2 ICL in vPPNs (Fig. 5F). *y334-Gal4* fish expressing *UAS:ICL-GFP* showed reduced behavioral arrest (Fig. 5G), whereas controls that expressed a mutant form of the ICL that does not disrupt GABAergic signaling responded normally (Fig. 5G) (*39*). Together, these data indicate that GABAergic inhibition of *y334-Gal4* vPPNs is required to initiate behavioral arrest in response to an overwhelming threat.

### Prepontine arrest neurons increase activity following persistent flow stimuli

**Fig. 6.**
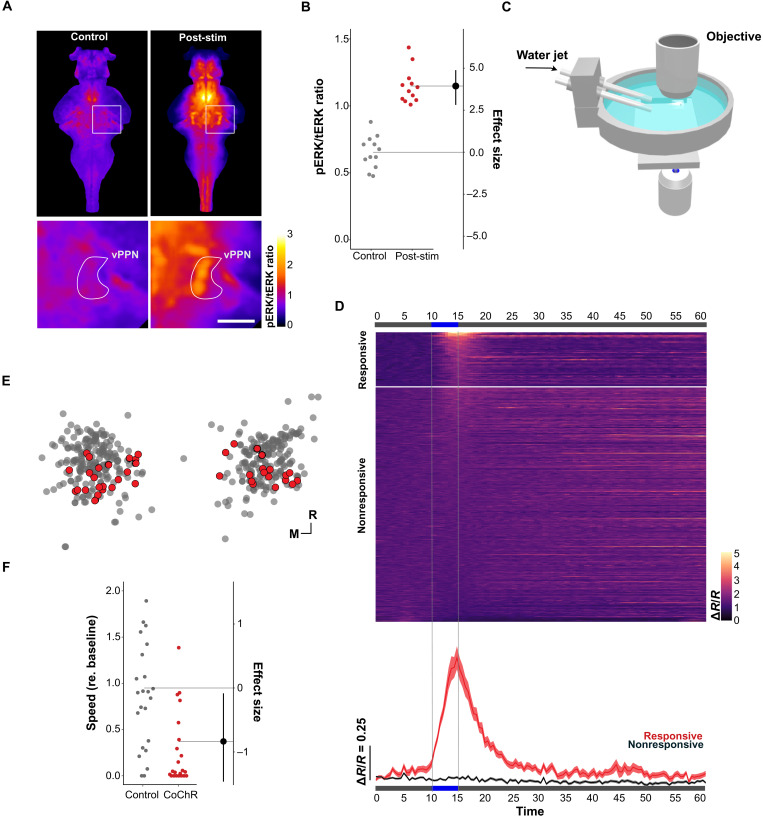
vPPN activation correlates with arrest induced by intense stimuli. (**A**) Dorsal plane from registered, averaged pERK/tERK ratios in control (*n* = 12 fish, left) and vibration-exposed fish (*n* = 12, right). Color shows p/tERK ratio (AU). Bottom: Magnified region around right vPPN. Scale bar, 30 μm. (**B**) pERK/tERK ratios in vPPN masked regions for control (gray, *n* = 12) and vibration stimulus-exposed (red, *n* = 12) conditions. ANOVA *F*_1,22_ = 92.98, *P* < 0.001. (**C**) Apparatus used to deliver pulsed water jets at head-fixed zebrafish during calcium imaging. (**D**) Top: Activity in flow-responsive (*n* = 41 neurons from 14 fish) and nonresponsive (*n* = 379 cells) neurons. GCaMP6s fluorescence normalized to coexpressed nuclear-localized red fluorescent protein (RFP; Δ*R*/*R*). Vertical lines show flow stimulation period. Bottom: Average ± SEM normalized GCaMP6s fluorescence for responsive (red) and nonresponsive (black) neurons. (**E**) Location of responsive (red) and nonresponsive (gray) neurons within the vPPN. (**F**) Change in speed relative to baseline (mm/s) in sibling controls (gray, *n* = 23 fish) and *y334-Gal4*, *UAS:CoChR-tdTomato* (red, *n* = 22 fish) following 15 s, 60 Hz (50% duty cycle) photostimulation. Effect size is Cohen’s *d*. ANOVA *F*_1,45_ = 8.196, *P* = 0.006.

To test whether vPPNs respond to threat, we measured changes in phosphorylated extracellular signal–regulated kinase (pERK), a marker for neural activity, using immunohistochemical labeling after exposure to the vibratory stimulus (*40*). Stimulation led to increased pERK/tERK ratios in the posterior tuberculum, midbrain tegmentum, and prepontine tegmentum including in the area occupied by vPPNs (Fig. 6, A and B). However, because changes in pERK lag neuronal activity by around 2 min (*41*), it was possible that the pERK signal reflected peri-stimulus activation preceding behavioral arrest. We therefore examined vPPN activity at higher time resolution by expressing a nuclear localized *UAS:GCaMP6s* in *y334-Gal4* and measuring changes in fluorescence after pulsed mechanosensory stimulation in a head-fixed preparation (Fig. 6, C to E). Because it was not feasible to deliver vibratory stimuli during imaging, we used a repeated pulsed jet stimulus paradigm. Like vibratory stimuli, water jets evoke robust startle responses (*42*) and, therefore, repeated stimuli simulate an inescapable stimulus. A subset of vPPNs (*n* = 41 of 420 neurons from eight fish) showed a strong increase in fluorescence after 5 s of stimulation (Fig. 6, D and E). Activity in stimulus-responsive cells ramped slowly, with fluorescence peaking at the cessation of the stimulus. The delayed increase cannot be accounted for by the relatively slow kinetic profile of nuclear-localized GCaMP6s, which peaks ~1.1 s after activation (*43*) and, therefore, suggests that vPPN activity builds during exposure to an intense mechanosensory stimulus.

Because vPPNs were active during arrest-inducing stimuli, we reasoned that triggering vPPN firing would evoke arrest in the absence of vibrational stimuli. To test this, we optogenetically activated *y334-Gal4* vPPNs using the channelrhodopsin variant *UAS:CoChR-tdTomato* (*44*). Although spontaneous activity was unchanged under baseline conditions, photoactivation in free swimming *y334-Gal4*, *UAS:CoChR* fish (Fig. 6F) resulted in locomotor suppression after photoactivation similar in magnitude to that produced by intense vibration compared to sibling controls lacking *UAS:CoChR*, which showed no change in motor activity (Fig. 6F). Thus, consistent with vPPN ablation eliminating arrest, *y334-Gal4* vPPN activation simulated exposure to an intense threat and induced arrest.

### Prepontine neurons contact premotor reticulospinal neurons

**Fig. 7.**
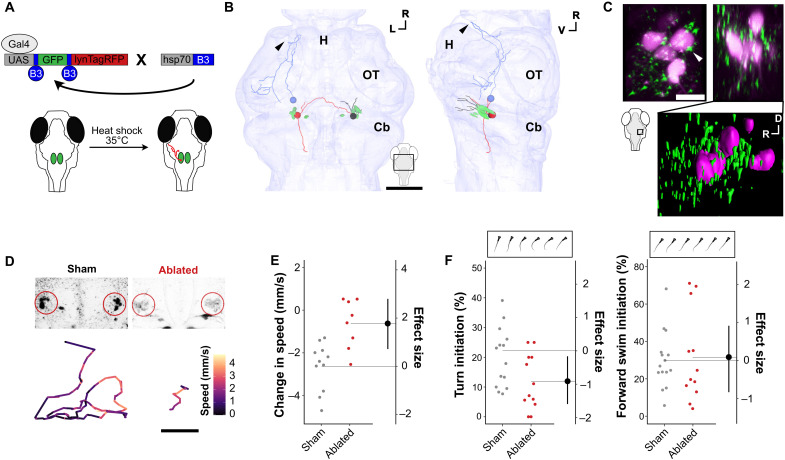
vPPN drive arrest via RoL1 premotor neurons. (**A**) Schematic of intersectional method for sparse labeling of single neurons with membrane-tagged RFP (lynTagRFP) to enable morphological reconstruction. (**B**) Reconstructed *y334-Gal4* vPPNs and RoL1 neurons (green) vPPNs project to ipsilateral (black), contralateral (red), and hypothalamic (blue) targets. Arrowhead: terminal projection in rostral hypothalamus. All neurons can be viewed in fig. S7. Scale bar, 50 μm. H, hypothalamus; OT, optic tectum; Cb, cerebellum. (**C**) Top: Maximum projection of *y334-Gal4*;UAS:synaptophysin-RFP (green) and backfilled RoL1 (magenta) colocalization in dorsal (left) and lateral (right) views. White arrowhead: apposition of *y334-Gal4* synapses with RoL1 cell body. Scale bar, 10 μm. Bottom: 3D rendering of lateral view showing synapses relative to RoL1 and surrounding neuropil. (**D**) Top, left: Sham-ablated and (right) multiphoton-ablated RoL1 reticulospinal neurons. Bottom: Baseline movement traces for sham-ablated (left) and RoL1-ablated (right) fish. Color denotes speed within a 5-s epoch. Scale bar, 1 cm. (**E**) Change in speed (mm/s) after vibratory stimulus in sham-ablated (gray, *n* = 11 fish) and bilateral RoL1-ablated (red, *n* = 8 fish). Effect size is Cohen’s *d*. *t* test, *P* = 0.001. (**F**) Turn initiation (left) and forward swim initiation. *t* test, *P* = 0.03. Right: Frequency during baseline locomotion in sham-ablated (gray, *n* = 14) and RoL1-ablated (red, *n* = 12) fish. Insets show examples of turn and forward swim bouts.

The critical role of vPPNs in evoking arrest led us to map efferent targets of vPPNs. We labeled individual *y334-Gal4* neurons using an intersectional approach combining *UAS:blo-GFP-blo-lynTagRFP* and a heat shock–inducible B3 recombinase, taking advantage of the inefficiency of B3 recombinase in zebrafish to facilitate sparse red fluorescent protein (RFP) labeling by excising the *GFP*-stop cassette between its cognate blown-out (blo) sites (Fig. 7A) (*45*). Morphological reconstruction of 13 neurons (*n* = 5 fish) revealed three classes of vPPNs (Fig. 7B). The first class (seven neurons) projected to the ipsilateral ventral neuropil. We noted that these projections passed within the margin of registration error to cell bodies of RoL1 neurons, a cluster of reticulospinal neurons that drive forward swimming and stimulus-evoked turns (Fig. 7B) (*46*, *47*). A second class of vPPNs (five neurons) projected ventrally past ipsilateral RoL1s and also crossed the midline to terminate near the contralateral RoL1 cluster. The third class (two neurons) projected anteriorly to the hypothalamus and torus semicircularis. To test whether vPPN termini contacted RoL1 neurons, we expressed *UAS:synaptophysin-RFP* in *y334-Gal4* and backfilled RoL1 neurons using fluorescently labeled dextran injected into the spinal cord. Fluorescent puncta from vPPN presynaptic terminals colocalized with RoL1 cell bodies (Fig. 7C). We also observed numerous presynaptic puncta in the neuropil region surrounding RoL1 neurons.

To test whether RoL1s were part of the arrest pathway, we ablated them using a multiphoton laser before testing for changes in locomotor behavior and vibration-evoked arrest. We confirmed previous reports that RoL1 ablation reduced baseline locomotor behavior (Fig. 7, D and E, and fig. S8) (*46*, *47*). RoL1-ablated fish also showed a loss of behavioral arrest following vibratory stimuli (Fig. 7F and fig. S8A). Analysis of swim path trajectories showed that RoL1 ablation not only suppressed overall displacement but also resulted in straight-line swim trajectories similar to those observed during vibration-induced arrest (Fig. 7D). Kinematic analysis of movement using high-speed video recordings showed that RoL1 ablation led to a specific reduction in turn bouts, with forward swims relatively spared (Fig. 7F). These results support a model in which RoL1 neurons promote baseline locomotion that is suppressed via vPPNs in response to severe threat.

## DISCUSSION

**Fig. 8.**
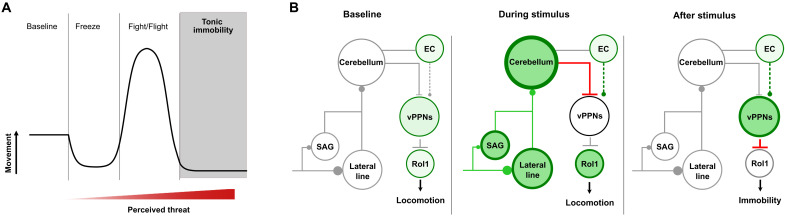
Circuit model for tonic immobility. (**A**) Defense cascade behaviors represented in terms of relative movement with respect to perceived threat. (**B**) Repeated, inescapable stimuli activate peripheral auditory (SAG) and lateral line ganglia, which then excite the granule cells of the cerebellum (green lines). During stimulus presentation, the cerebellum sends inhibitory projections to the prepontine neurons (vPPNs) and eurydendroid cells (ECs), which may project to vPPNs (dashed lines). Following sustained vibratory stimulation, cerebellar inhibition ceases and leads to activation of the vPPNs. Bilateral activation of the vPPNs disrupts RoL1 neuron activity, leading to suppressed turning behavior and immobility.

Understanding how animals perceive threatening stimuli and rapidly initiate defensive responses is fundamental for understanding cognitive states such as fear. Our data reveal that larval zebrafish, when presented with an intense and inescapable threat stimulus, respond with behavioral arrest with characteristic features of tonic immobility. As observed in mammals, reptiles, and birds, tonic immobility in zebrafish manifests as an innate response defined by locomotor suppression with abrupt recovery, lack of responsiveness to external stimuli, and delays in the righting reflex. Given that tonic immobility follows inescapable threat stimuli, our data suggest that the defense cascade framework may be conserved across vertebrates (Fig. 8A). Moreover, the magnitude and persistence of tonic immobility in zebrafish is intensity dependent as described in chick and lizards (*6*, *48*). We note that tonic immobility is often accompanied by other behavioral and physiological correlates that we did not measure, including decreased heart rate, increased respiration, tremor, and changes in correlated neural activity (*7*, *19*). Further investigation will elucidate whether zebrafish exhibit these physiological changes and may reveal further core conserved elements of this behavior.

On the basis of our data, we propose a sensory afferent-cerebellar-prepontine circuit in which vPPNs are crucial for initiating arrest following an intense threat (Fig. 8B). In this model, at baseline, vPPNs show low levels of activity, allowing RoL1s to propel normal locomotor activity. During an intense vibration, persistent lateral line and auditory/vestibular inputs to granule cells together increase cerebellar activity. After the cessation of the stimulus, loss of GABAergic signaling from Purkinje cells elicits increased firing in vPPNs via rebound that, in turn, suppress RoL1 firing and disrupt swimming. This hypothetical model describes a circuit by which inescapable threatening stimuli induce behavioral arrest in zebrafish and may serve as a model for conserved pathways that mediate tonic immobility in other species.

In our proposed working model, persistent Purkinje cell inhibitory signaling elicits vPPN activation. These data agree with previous studies demonstrating a loss of neck-pinch evoked tonic immobility in *cerebelless* mouse mutants, which lack GABAergic neurons in the cerebellar nuclei (*49*). How does increased inhibitory signaling cause increased vPPN activity? Electrophysiological recordings from Purkinje cell targets show that increased inhibitory postsynaptic potentials (IPSPs) correlated with increased firing rates (*37*, *50*). Purkinje cell IPSP-driven increase in firing rate requires synchronized input from multiple Purkinje cells, which entrain downstream targets to fire synchronously (*50*). Pharmacological GABA facilitation and *y334-Gal4*–specific GABA_A_ receptor blockade both disrupted vibration-evoked behavioral arrest (Fig. 5, C to E). Although these results appear contradictory, we propose that disrupting GABAergic signaling with *UAS:ICL* blocks Purkinje cell inputs to vPPNs whereas pharmacological GABA agonism disrupts synchronized Purkinje inputs onto vPPNs. However, GABA agonism through local injections into tegmental structures such as the periaqueductal gray and dorsal raphe also reduces tonic immobility in mammals, suggesting that multiple tegmental structures may also be involved in regulating tonic immobility (*51*).

Our model is limited by our incomplete understanding of direct synaptic and functional connectivity between Purkinje cells and other cerebellar output neurons to vPPNs in zebrafish. For example, the glutamatergic eurydendroid cells, which are analogous to the mammalian deep cerebellar nuclei, may also plausibly project to vPPNs as a source of cerebellar excitation (Figs. 5C and 8B). However, Purkinje neurons directly project to the parabrachial complex in the prepontine region of mouse and rabbit (*52*), suggesting that this connection may be functionally relevant. We propose that increased Purkinje cell inhibition and/or excitatory drive from eurydendroid cells driving vPPN activation is a plausible mechanism for arrest and invites future experiments to explicitly test this hypothesis.

How does bilateral activation of glutamatergic vPPNs result in locomotor inhibition? vPPNs project bilaterally to RoL1 reticulospinal neurons (Fig. 7, B and C) and bilateral RoL1 ablation leads to a decrease in activity similar to vPPN activation (Fig. 7, D to F). vPPNs show strong expression of several stress-associated neuropeptides including *crhb*, *trh*, and *uts1*. Corelease of these neuropeptides with glutamate may suppress or disrupt RoL1 function, leading to behavioral arrest and potentially accounting for the prolonged duration of immobility after vPPN activation. Alternatively, vPPNs may modify other inputs to RoL1 neurons; RoL1s are located in a dense neuropil that receives projections from many brain regions (*46*). vPPNs make synaptic contacts within this neuropil (Fig. 7C) and may, therefore, influence other convergent pathways that influence swim initiation.

Our data define a previously unidentified region within the zebrafish prepontine tegmentum that mediates responses to persistent threat. Neurons in the vPPN form a characteristic crescent shape in both *y334-Gal4* and *y405-Gal4* transgenics, and these lines have largely overlapping expression within this domain. RNA sequencing from *y334-Gal4* and *y405-Gal4* and in situ hybridization experiments disclosed that vPPNs in both lines are enriched in *crhb*, the teleost homolog of *corticotropin-releasing hormone* (*CRH*), which is consistent with previous in situ hybridization studies that revealed *crhb* expression in the anterior hindbrain (*53*). CRH is a prominent regulator of the hypothalamus-pituitary-adrenal axis, and indeed, *CRH* receptor activation in rodent amygdala and periaqueductal gray facilitates tonic immobility (*54*). Moreover, *y334-Gal4* cells expressed *CGRP* (calca) and were enriched in *spexin* (*spx*), a neuropeptide implicated in anxiety, energy homeostasis, and nociception, and *urotensin* (*uts1*) and *thyrotropin-releasing hormone* (*trh*), which are part of the hypothalamic-pituitary-interrenal axis and are implicated in stress responses (*55*, *56*). The roles of these neuropeptides in mediating tonic immobility remain an open and intriguing question.

vPPNs are bounded by the locus coeruleus and nucleus isthmi (the teleost homolog of the parabigeminal nucleus; Fig. 4E). In mammals, a similar neuroanatomical region comprises the parabrachial complex, a structure in the prepontine region lateral to the locus coeruleus that surrounds the superior cerebellar peduncles (*34*). Like vPPNs, most parabrachial neurons are glutamatergic and are enriched in CRH expression (*34*, *57*). Moreover, as noted above, like vPPNs, the mammalian parabrachial receives a small direct input from cerebellar Purkinje neurons. The parabrachial nucleus integrates aversive stimuli and serves as a general alarm system for threats (*34*). Cholinergic stimulation of the parabrachial nucleus prolonged the duration of tonic immobility in guinea pig (*58*). Thus, based on similarity of position, function, and gene expression, we suggest that vPPNs are homologous to part of the mammalian parabrachial complex. Future experiments examining molecular markers of the parabrachial subnuclei and functional connectivity may provide more direct evidence for homology. If so, our data would indicate that the parabrachial complex has a deep evolutionary history in promoting defensive responses.

The cerebellar-vPPN structure was not involved in shock-mediated freezing behavior. Previous work has implicated the habenula as a locus for freezing and passive responsiveness in zebrafish and rodents (*16*, *59*, *60*). However, *y334-Gal4*–ablated larvae showed no changes in shock-mediated freezing (fig. S4) and Gal4 lines with habenular expression showed no effect on vibration-induced arrest. These differences suggest that vibration-induced tonic immobility and shock-induced freezing are controlled by independent circuits. Cerebellar lesions in rats result in decreased freezing behavior to a predator cue, but not to electrical shock (*61*). These two classes of stimuli are thought to be integrated in the hypothalamus and amygdala, respectively (*62*), supporting the idea of parallel defensive pathways. However, given that the cerebellum and parabrachial nuclei are also implicated in freezing (*63*, *64*), understanding how these regions influence the expression of two behaviors at opposite ends of the defense cascade remains an interesting question.

Defensive behaviors associated with fear are instances of “emotion primitives,” a conceptual framework that defines internal states with behavioral characteristics that scale with intensity, valence, and persistence (*65*). This study shows that tonic immobility in larval zebrafish meets these criteria and outlines the underlying circuit. Our data disclose that vPPNs play a central role in behavioral state control by activating the terminal behavior in the defense cascade. Understanding how the vPPNs override expression of other defensive behaviors will help us better understand how animals assess risk and match defensive responses to perceived threat.

## METHODS

### Animals and transgenic lines

All lines used in this study were maintained in a Tupfel Longfin (TL) background. The full list of transgenic lines used in this study is described in the Supplementary Methods. All experiments were approved by the NICHD ACUC.

Photoactivation experiments used plasmid pTol1-UAS:CoChR-tdTomato (a gift from C. Wyart, ICM) (*44*). The ICL and mutant ICL (ICLmut) constructs were generated by inserting a zebrafish codon-optimized geneblock [IDT; sequence from (*39*)] into *UAS:dusp27-GFP* (*66*) by replacing *dusp27* with ICL using restriction enzyme digestion and ligation. Plasmids were injected with tol1 transposase and screened for fluorescence before testing.

### Behavioral experiments

All behavioral experiments were conducted at 27° to 28°C. For initial behavioral arrest characterization, zebrafish larvae (6 to 7 dpf) were placed individually in 1 cm^2^ wells of a 3 × 3 arena and allowed to acclimate to ambient light for >2 min. The arena was lit from below with a white-spectrum light-emitting diode (LED) array; for experiments conducted in the dark, an infrared LED was used. Sinusoidal stimuli were generated using a BNC-2110 DAQ board (National Instruments) and delivered using a Type 3810 minishaker (Bruel-Kjaer). Vibratory stimuli were calibrated so that single stimuli evoked escape responses with 96% ± 3% SEM (*n* = 8 fish). Behavioral responses were captured at 20 frames per second using a μEye camera (IDS Imaging) fitted with a 40-mm macro lens (EX DG Macro, Sigma-Global). Each trial contained a 120-s baseline period followed by ~15 s of vibratory stimulation and a 210-s recovery period. Fish were imaged during the baseline and recovery period, but not during stimulation as clear images could not be obtained because of the intense vibration. Recording began 500 ms after stimulus termination, which allowed time for residual water movement and passive drifting to cease. Behavioral responses were analyzed using FLOTE software (*67*). Locomotion was measured as the total (*x,y*) distance moved within 5-s bins and fish that displayed an average speed of <0.5 mm/s during the baseline period were excluded from analysis. In cases where baseline locomotion was affected by experimental manipulation, we compared immobility only in fish with baseline locomotion between 2 and 3 mm/s under both conditions.

Half-recovery time was calculated as the time to recover to half of the baseline. Individuals that never recovered to half of the baseline were coded as having recovery times of 210 s. Locomotor path analysis was conducted with locomotion data sampled at 20 Hz. After smoothing position data using a moving average, we calculated the vector angle between consecutive points using the formula $\emptyset ={\text{tan}}^{-1}(\frac{y}{x})$.

Locomotor behaviors and escape responses were imaged using the same apparatus. Responses were recorded at 1000 frames per second using a DRS Lightning high-speed camera (DEL Imaging) and analyzed using FLOTE. Locomotor kinematics were measured in 400-ms epochs and categorized as turns or forward bouts using kinematic parameters described previously (*68*). Turn or forward bout initiation was calculated as the percent of epochs in which that bout was performed. For all epochs, only the first behavior was used for analysis.

Righting reflex was tested using the same apparatus used for high-speed video tracking. Groups of five fish were placed in a 5 cm by 5 cm arena and presented with a 15-s low-frequency stimulus to induce behavioral arrest; control fish were not stimulated. After stimulus presentation, all fish were presented with a 1-s high-amplitude, low-frequency stimulus to disrupt balance. Groups of fish were tracked at 1000 frames per second for 15 s. Righting was annotated manually by a blinded observer who recorded the proportion of unbalanced fish at the end of 500-ms epochs. “Time to balanced” was defined as the time at which all five fish in the arena showed a dorsal-up posture.

Acoustic and electric field–evoked startle escape response experiments were conducted as previously described (*25*). Acoustic (800 Hz, 5 ms sinusoidal wave) or electric (2 V/cm, 2 ms square wave) pulse stimuli were delivered at 30-s intervals to minimize habituation and categorized as short-latency or long-latency startle responses using kinematic and response latency parameters. Responsiveness was calculated as the percent of responses under each condition (baseline, arrest, and recovery). For dark flash responses, larvae were illuminated from above with white-spectrum light (75 μW/cm^2^) for the duration of the experiment. Dark flash stimuli consisted of a total loss of illumination for 300 ms and were delivered at 60-s intervals. Responses were characterized using kinematic parameters and responsiveness was calculated in the same manner as the SLC experiments.

### Ablations

Chemogenetic ablations were conducted according to previously published protocols (*27*, *45*). Gal4 lines were chosen for the circuit-breaking screen using the zebrafish brain browser (zbbrowser.com), crossed with *UAS:epNTR-TagRFP*; embryos were screened for TagRFP fluorescence at 3 to 4 dpf. In Gal4-Cre experiments, fish were screened for the presence of RFP in the predicted intersectional expression pattern. Nonfluorescent sibling embryos were used as controls in both cases. Both groups were treated with 10 mM metronidazole (Sigma-Aldrich) in dim light (27 μW/cm^2^) for 24 to 48 hours. Following ablation, larvae were washed in E3 and allowed to recover for >12 hours before testing. Following behavioral experiments, a subset of ablated fish was inspected using epifluorescent microscopy to confirm full pattern ablation.

Neuromast ablations were conducted using 200 μM neomycin in E2 embryo medium according to (*69*). Six days post-fertilization larvae were immersed in neomycin for 1 hour, rinsed four times in fresh E2, and allowed to recover for 3 to 6 hours in E2 before testing. Following testing, ablation efficiency was confirmed using the fluorescent dye 2-4-(dimethylamino)styryl-*N*-ethylpyridinium-iodide (DASPEI; Sigma-Aldrich). Larvae were immersed in 0.005% DASPEI in E3 for 15 min and then rinsed twice in E3. Larvae were imaged using epifluorescent microscopy with a 488-nm filter to confirm ablation.

Multiphoton laser ablations were conducted on 4 dpf larvae raised in 300 μM *N*-phenylthiourea (PTU) in E3 to suppress melanin formation. PTU was added ~24 hours post-fertilization (hpf) and changed every 48 hours. Larvae were sorted for *UAS:kaede* expression at 3 dpf. Larvae were anesthetized in MS222 and mounted in 2.5% low–melting point agarose. Ablations were performed on a Leica TCS SPII upright confocal microscope with a MaiTai DeepSee multiphoton laser (Spectraphysics) using a 20×/1.00 NA (numerical aperture) water immersion lens. Single cells within the Gal4 pattern were identified visually using a 488 nm laser and ablated using a pulsed 800- to 850-nm tuned multiphoton beam. Ablations were confirmed visually using the 488-nm laser and transmitted light. Sham ablation animals were mounted in the same manner but were exposed to low laser intensity. Ablated and sham control larvae were raised in E3 until behavioral testing at 6 dpf and laser ablations were confirmed by confocal microscopy after behavioral testing.

### Drug exposure experiments

Ethanol and GABA exposure experiments were conducted on 6 dpf fish as described above. Stock solutions of 1 mM GABA and 50% ethanol were diluted in E3 to the working concentration. Fish were immersed for 20 min in solution immediately before the experiments and controls were placed in an equal volume of E3. In the ethanol experiment, fish were immersed in 300 mM ethanol for 20 min and fish designated for the washout experiment (*n* = 27 fish) were rinsed twice in E3 and placed into fresh E3 for an additional 20 min before testing.

### Cell capture and RNA sequencing

Cells from *y334-Gal4*;*UAS:kaede* and *y405-Gal4*;*UAS:kaede* were collected using a modified protocol from (*70*). Prepontine regions from 5 to 6 dpf fish were dissected into 134 mM NaCl, 2.9 mM KCl, 2.1 mM CaCl_2_, 1.2 mM MgCl_2_, 10 mM glucose, and 10 mM HEPES (pH 7.5) with NaOH and dissociated in neutral protease (1 mg/ml; Worthington) in Evans buffer for 30 min at room temperature with gentle shaking. The tissue fragments were rinsed three times with phosphate-buffered saline and triturated. Dissociated cell suspensions were plated onto a Sylgard-coated petri dish. Kaede-expressing cells were aspirated into a glass micropipette under a fluorescence microscope. Collected cells were visually verified by plating into a fresh dish and then the process was repeated until pure samples of kaede-expressing cells were obtained. Groups of 6 to 10 cells were dispensed from the micropipette into 1 μl of ice-cold 10× reaction buffer (SMART-Seq v4 kit, Takara) and flash-frozen before library preparation. Control samples were 50 to 100 nonprepontine cells. cDNA synthesis was performed using the SMART-Seq v4 Kit according to the manufacturer’s instructions. After cDNA purification (Beckman SPRI beads), quality control (Agilent Bioanalyzer), and quantification, samples were sequenced on an Illumina Novaseq with paired end reads. Raw read counts were conormalized using DESeq in R (*71*) and genes with expression <5000 reads in vPPN samples were filtered to remove low-expression genes. Differential expression was calculated using nonparametric Wilcoxon Rank-Sum tests and *P* values were corrected for multiple comparisons using a Benjamini-Hochberg correction with a 5% false discovery rate.

### Optogenetic activation experiments

Transgenic Gal4-expressing embryos were injected at the one-cell stage with 10 ng/μl of a plasmid containing *UAS:CoChR-tdTomato* with tol1 mRNA (80 ng/μl) and raised in E3 under low-light conditions to reduce blue light exposure. Larvae were screened for RFP fluorescence at 3 dpf and injected embryos without RFP expression were used as controls. Behavioral experiments were conducted on the same apparatus as the vibration experiments with a 470-nm LED (Prizmatix) replacing the minishaker. Experiments were done with IR illumination in dim light (30 μW/cm^2^). Following a 10-min acclimation period, larvae were presented with a 15-s 60-Hz pulsed stimulus (650 μW/cm^2^) with a 50% duty cycle to simulate the vibratory stimulus, and behavior was recorded and analyzed as described earlier.

### Imaging

Larvae were raised in PTU beginning at 24 hpf. For whole-brain images, 5 dpf larvae were immersed in a solution of 0.001% Lysotracker DeepRed (Invitrogen) in 1% dimethyl sulfoxide in E3 for 12 to 18 hours and then rinsed in E3 twice before imaging. At 6 dpf, larvae were anesthetized in MS222 and dorsally mounted in 2.5% low–melting point agarose within Lab-Tek II #1.5 cell culture chambers. In some cases, kaede was photoconverted from green to red using 405-nm light (50 μW/cm^2^) for 10 min before imaging. Whole and partial brain images were acquired on a Leica SPII inverted confocal microscope with a 25×/0.95 NA water immersion lens. Images were acquired at 1 μm by 1 μm by 2 μm voxel resolution. Samples were excited using a 488-nm argon laser and a 561-nm solid-state laser and detected using hybrid detectors for GFP and RFP channels and a PMT detector for far red fluorescence. Images were postprocessed for dye separation using Leica Application Suite software. Three-dimensional rendering of imaged fish was conducted in Imaris 8.4.2 (Bitplane).

Individual neurons were labeled by crossing *y334-Gal4*;*UAS:blo-Switch* fish to *hsp70:B3* and raised in PTU. Individual neurons were labeled at 3 dpf by a 20-min heat shock at 35°C to drive expression of B3 recombinase. Larvae were imaged at 6 dpf and axon projections were traced semi-automatically in Imaris using the filaments feature.

Whole-brain images were acquired using 616 × 500 pixel tiles with 25-μm overlap and stitched post hoc using custom Fiji scripts. Images were then registered using Advanced Normalization Tools (*72*, *73*). Registered images were masked to remove autofluorescence from skin and the eyes.

pERK/tERK immunohistochemical labeling was conducted similar to previously published protocols (*40*). Briefly, TL fish were raised in PTU until 6 dpf. Fish were presented with arrest-inducing stimuli and fixed in 4% paraformaldehyde (PFA) 2 to 5 min after stimulus presentation. Control fish were exposed to the vibratory stimulus but fixed >10 min after stimulus presentation. Fixed fish were labeled using immunofluorescence with pERK (Phospho-pp44/42 MAP Kinase) and tERK (pp44/42 MAP Kinase) antibodies (Cell Signaling Technologies) and Alexa fluorophore–conjugated secondary antibodies at 1:500 dilutions. After registration to the tERK pattern in the Zebrafish Brain Browser (zbbrowser.com), images were masked using a vPPN mask generated from a binarized *y334-Gal4* pattern. pERK/tERK ratios were calculated and averaged within the masked area for each imaged fish.

Fluorescence in situ labeling for *crhb* and *calca* was performed using HCR (*74*). Split initiator probe sets against *crhb* (Accession number NM_1001007379) and *calca* (Accession number NM_001002471) with B3 initiation sequences were purchased from Molecular Instruments with B3-488 nm–conjugated hairpins. *y405-Gal4*;*UAS:kaede* fish were raised in PTU and fixed in 4% PFA at 6 dpf. Following fixation, kaede was photoconverted as above. HCR was performed on whole-mount embryos according to the manufacturer’s instructions and imaged using confocal microscopy. Colocalization counts were performed using FIJI.

Visualization of reticulospinal neurons was conducted by backfills of *y405-Gal4* and *y334-Gal4* with *UAS:kaede*, *UAS:LynTagRFP*, or *UAS:eGFP-CAAX* raised in PTU. Five days post-fertilization larvae were anesthetized in MS222 and placed into a Sylgard-coated dish. A ~50% solution of either 3000 MW tetramethylrhodamine biotinylated dextran or 10,000 MW Alexa 488–conjugated dextran (Molecular Probes) was injected into the spinal cord immediately dorsal to the swim bladder using a PV820 pneumatic picospritzer (World Precision Instruments). Larvae were allowed to recover in Evans buffer for 24 hours and imaged as described above.

### Calcium imaging

Calcium imaging was performed on fish injected with tol1 mRNA (80 ng/μl) and *UAS:nls-GCaMP6s-2a-nls-dsRed* plasmid (10 ng/μl) at the single-cell stage. Larvae were raised in PTU and screened for dsRed fluorescence at 3 dpf. At 6 dpf, larvae were mounted in 2.5% low melting point agarose in a 35-mm^2^ petri dish. The agarose surrounding the tail was removed to allow access to water pulses. Mounted larvae were placed in a custom 3D-printed stage on a Leica SPII upright confocal microscope with a resonant scanner and a 20×/1.00 NA water dipping lens. Pulsed stimuli were delivered using a Cole-Parmer self-priming micropump controlled by a BNC-2110 DAQ board. Each stimulus set consisted of a 10-s baseline period followed by 5-s stimulus presentation of 1-Hz water pulses and a 45-s recovery period. In experiments with multiple stimulus presentations, stimulus sets were separated by at least 120 s to minimize effects of consecutive stimuli. GCaMP6s activity was recorded using 488 nm and dsRed at 561-nm excitation at 2 Hz in single planes through vPPNs. GCaMP6s fluorescence was measured from image time series using custom Python scripts and the scikit-image toolbox. Nuclear position was identified from the dsRed channel using the Laplacian of Gaussian method of blob detection. Nuclear position was then registered using manual affine registration using the first 10 frames of the dsRed channel as a reference. Nuclear dsRed and GCaMP6s fluorescence was measured for each neuron, and Δ*R*/*R* was computed by dividing GCaMP6s by dsRed fluorescence. Then, normalized fluorescence was divided by the average baseline normalized fluorescence to obtain a final measure of fluorescence. Trials where cells drifted out of the *z*-plane or where the animal exhibited struggle behavior were discarded. Cells that increased fluorescence by >3 standard deviations (SD) above the mean during the stimulus period were characterized as stimulus-responsive cells.

### Statistics

Data were analyzed using custom scripts in IDL (Harris Geospatial), R (www.R-project.org), and Python 3.7. Estimation plots were generated using the dabestr library in R (*75*) and reported effect sizes are Cohen’s *d*, calculated as mean difference divided by pooled SD. All *t* tests are two-tailed and independent samples unless otherwise noted.

## Supplementary Materials

### This PDF file includes:

Supplementary Methods Figs. S1 to S8 References

### Other Supplementary Material for this manuscript includes the following:

Movie S1

Interactive 3D reconstruction of vPPNs

## Notes

View/request a protocol for this paper from *Bio-protocol*.
