# Supplementary material for: A cerebellar-prepontine circuit for tonic immobility triggered by an inescapable threat
Source: Sci Adv. 2022 Sep 28;8(39):eabo0549. doi: 10.1126/sciadv.abo0549 (PMC9519051; doi:10.1126/sciadv.abo0549)
Supplement: Supplementary file 1 — Supplementary Methods Figs. S1 to S8 References [file sciadv.abo0549_sm.pdf]

Science Advances
AAAS

Supplementary Materials for

A cerebellar-prepontine circuit for tonic immobility triggered by an inescapable threat

Ashwin A. Bhandiwad *et al.*

Corresponding author: Ashwin A. Bhandiwad, ashwin.bhandiwad@nih.gov;
Harold A. Burgess, burgessha@mail.nih.gov

Sci. Adv. **8**, eabo0549 (2022)
DOI: 10.1126/sciadv.abo0549

The PDF file includes:

Supplementary Methods
Figs. S1 to S8
Legend for movie S1
References

Other Supplementary Material for this manuscript includes the following:

Movie S1
Interactive 3D reconstruction of vPPNs

Supplementary Methods

Gal4 lines used in this study (*y256-Gal4*, *y318-Gal4*, *y334-Gal4*, *y397-Gal4*, *y405-Gal4*) have been reported previously (*27*). *aldoca:GFF (aldoca:Gal4)nub6Tg* (*76*) were a kind gift from Masahiko Hibi (Nagoya University). Gal4 lines were maintained with *Tg(UAS:Kaede)s1999t*. Nitroreductase lines *Tg(UAS-E1b:BGi-epNTR-TagRFPT-oPre)y268Tg (UAS:epNTR)* and *Tg(UAS:epNTR-TagRFPT-utr.zb3)y362Tg* were used for genetic ablation experiments (*25*, *27*). Gal4-Cre intersectional ablations were conducted using *Et(REx2-SCP1:BGi-Cre-2a-Cer)y520* and *Tg(14xUAS-E1b:BGi-lox-GFP-sv40-lox-epNTR-TagRFPT)y518* (*28*). Other UAS lines used in this study were *Tg(UAS:EGFP-CAAX)m1230* (*77*), *Tg(14xUAS-E1b:BGi-nls-GCaMP6s.zf1-2a-nls-dsRed2.zf1)y510* (*45*), *UAS:lynTagRFPT(y260)* (*20*), *Tg(UAS-E1b:BGi-SCN5a-v2a-TagRFPT)y266* (*25*), *and cUAS:PSD95.FingR-GPF-ZFC(CCR5TC)-KRAB(A)* (*36*).

Transgenic lines used for imaging neurotransmitter identity identification were *TgBAC(slc17a6b[vglut2a]:loxP-DsRed-loxP-GFP)nns14)*, *TgBAC(gad1b:LOXP-RFP-LOXP-GFP)nns26* (*78*), *vachta:GFP* (kind gift from Shin-ichi Higashijima, National Institute for Basic Biology, Japan), and *Tg(neurod1:nsfa-EGFP)vo4Tg* (*79*).

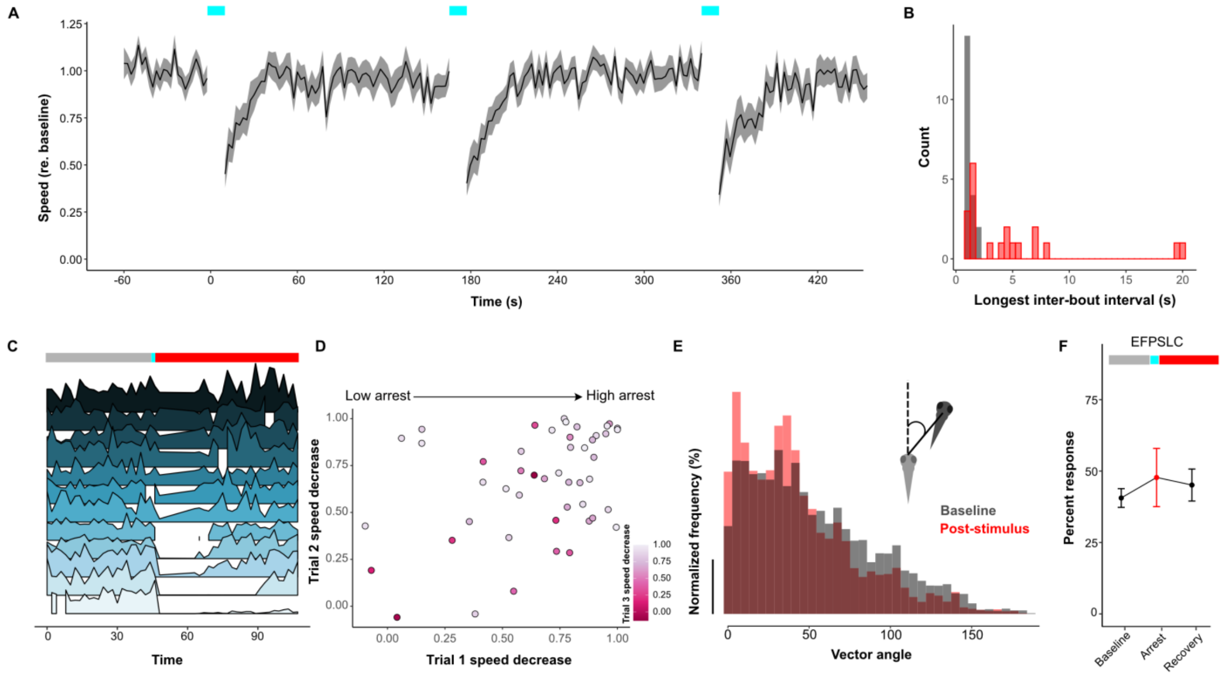

Fig. S1. Behavioral arrest is robust, replicable, and consistent within individuals
(**A**) Mean speed (relative to the average baseline speed) with repeated presentation of 26 dB re. 1 m/s^2 stimuli (cyan bars) (n = 27 fish). Shaded area for each trace is SEM. (**B**) Histogram of longest period of immobility during baseline (grey) and post-stimulus (red) conditions. (**C**) Displacement during 2 sec periods for individual fish at baseline and after the intense vibration. (**D**) Trial-to-trial variability shown as speed decrease in Trial 1 (x-axis) and speed decrease in Trial 2 (y-axis). Colors shows speed decrease in Trial 3; r^2 = 0.127 (**E**) Swim path vector angles during baseline (grey) and post-vibration stimulus (red). (**F**) Startle responsiveness to electric field pulses (EFP SLC, n = 27 fish) during baseline, arrest (red), and recovery periods (Mean $\pm$ SEM).

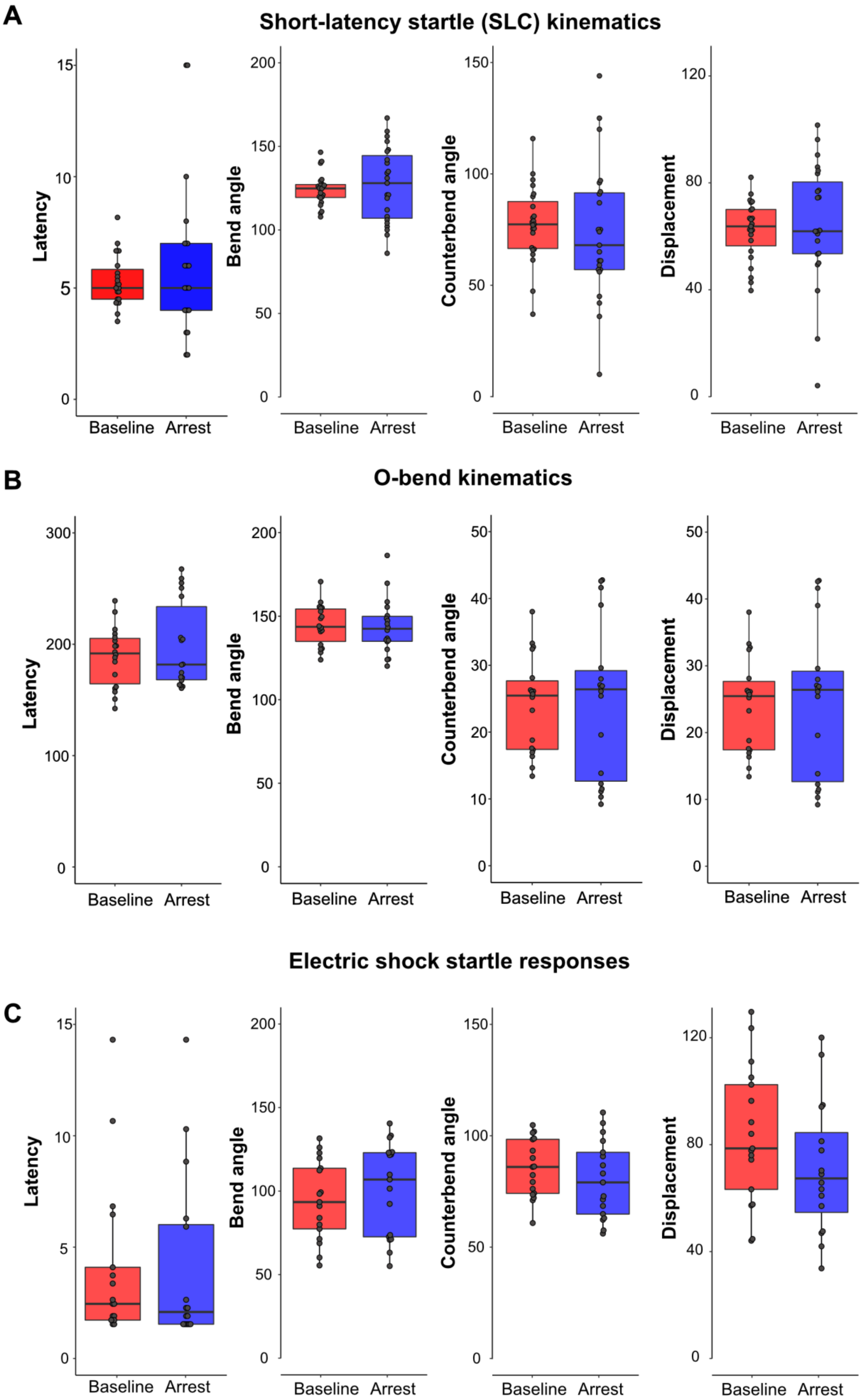
A
Short-latency startle (SLC) kinematics
Latency
Bend angle
Counterbend angle
Displacement
Baseline
Arrest
B
O-bend kinematics
C
Electric shock startle responses

Fig. S2. Startle and O-bend response kinematics are not altered during arrest

Kinematic parameters of short-latency startle responses to auditory cues (**A**), O-bend responses to dark flashes (**B**), and startle responses to electric fields (**C**) during baseline (red) and arrest (blue). Each data point represents mean data for a single fish (n = 27).

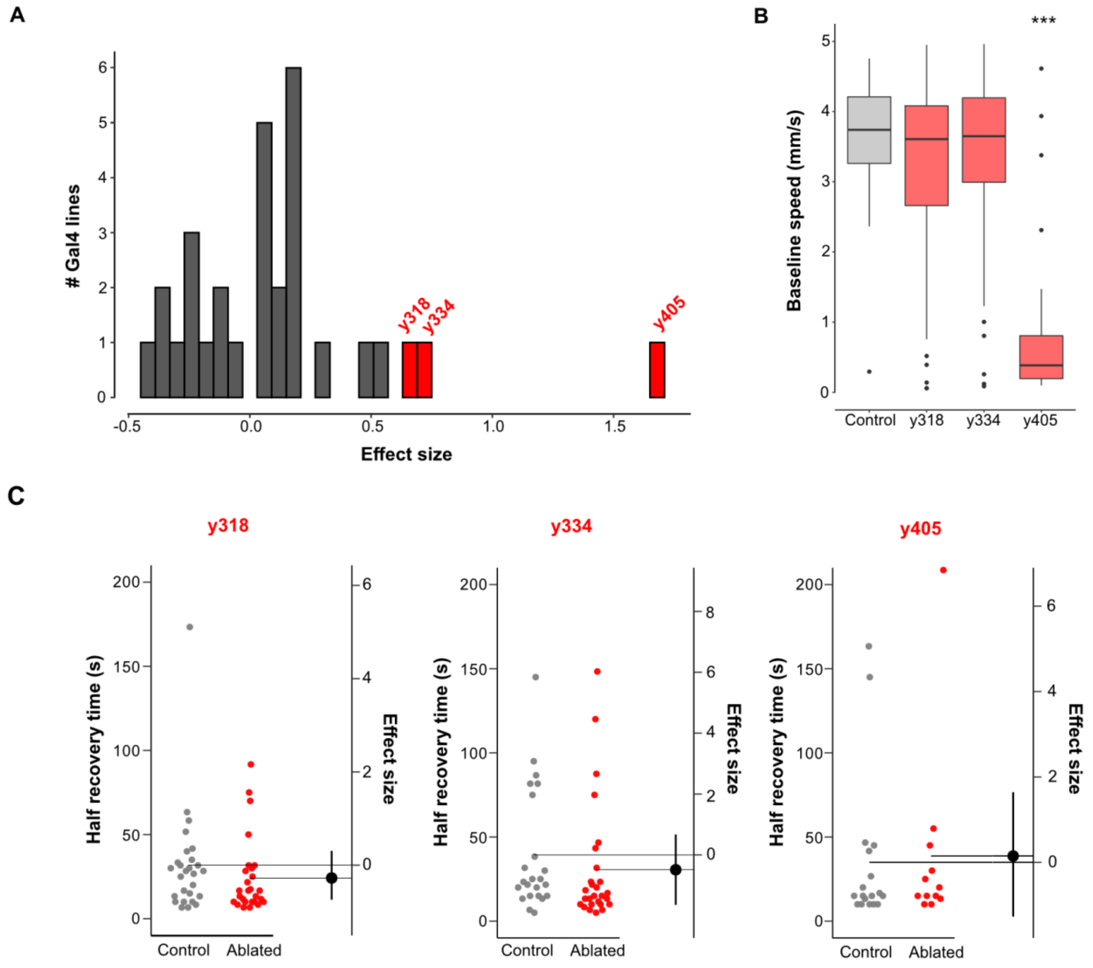

Fig. S3. Recovery time and baseline speed change for Gal4 lines implicated in arrest initiation

(**A**) Changes in vibration-stimulus evoked behavioral arrest for lines used in the circuit breaking screen. Data are Cohen's d for differences between control and ablated fish for each line. Lines with effect sizes > 0.6 are labeled. (**B**) Mean speed (mm/s) during the pre-stimulus baseline for control fish (grey), *y318-Gal4*, *y334-Gal4*, and *y405-Gal4*. t-test *** p< 0.001**.** (**C**) Time to half recovery (time to recover to 50% of pre-stimulus baseline speed) in control and ablated fish. No differences were statistically significant.

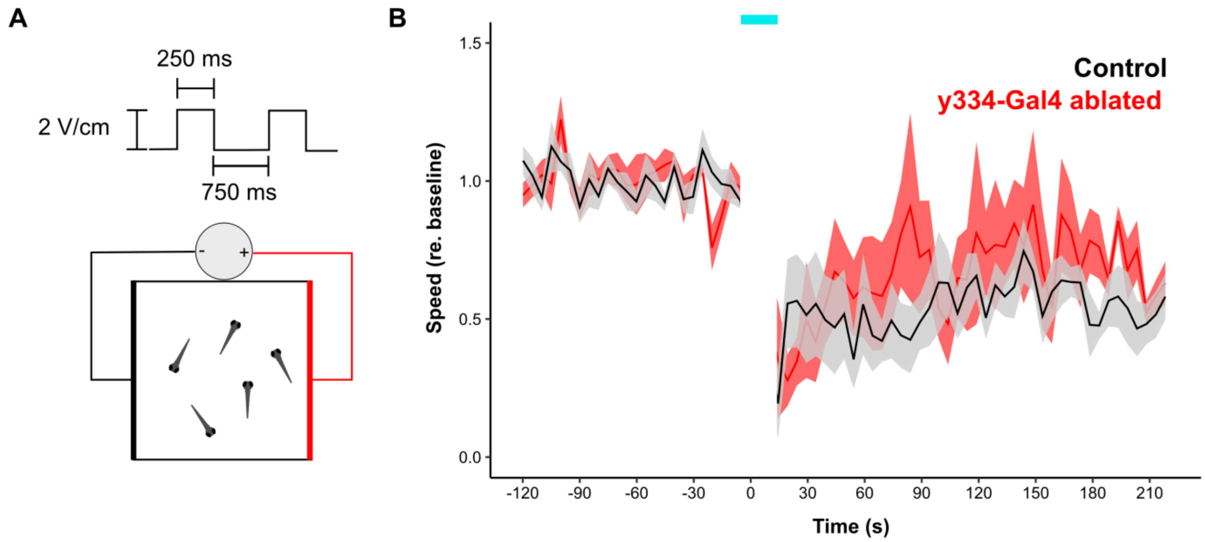

Fig. S4. *y334-Gal4* arrest does not generalize to electric shock stimuli

(**A**) Behavioral arena and stimulus parameters used for electric shock induced behavioral arrest. (**B**) Speed (relative to mean baseline) for control (black) and *y334-Gal4* ablated (red) fish after electrical field pulse stimulus. No differences were statistically significant (n = 15 fish). Shaded area represents SEM. Cyan bar shows stimulation period.

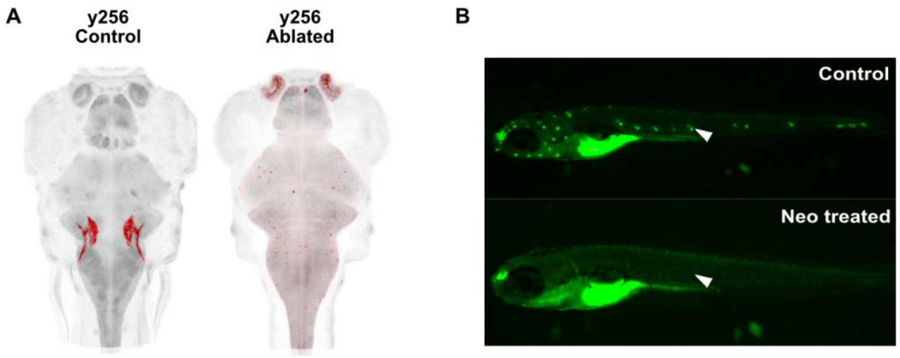

Fig. S5. Examples of statoacoustic ganglion and lateral line ablation

(**A**) *y256-Gal4* statoacoustic ganglion (SAG) ablation using NTR. Control (Left) and NTR ablated (Right) fish were imaged using the same laser settings at 7 dpf after behavioral testing and registered to a reference brain. (**B**) Lateral line ablation using 250 µM neomycin (Neo). DASPEI labeled neuromasts are visible in control (Top) and absent in Neo treated fish. Arrows show neuromasts

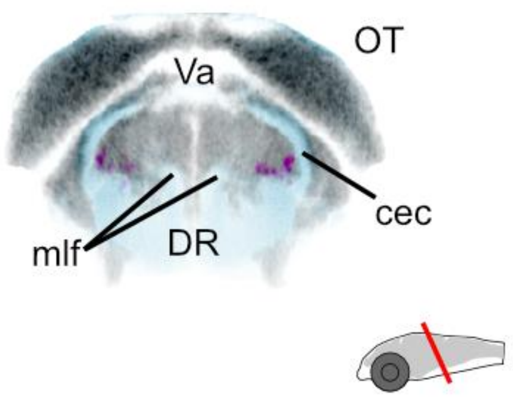

Fig. S6. vPPNs are located in ventral rhombomere 1

Oblique transverse section of 6 dpf larval zebrafish brain from the Zebrafish Brain Browser showing location of *y334-Gal4* vPPNs (magenta) in the prepontine tegmentum. Grey: soma locations (HuC-h2b-RFP). Blue: neuropil (HuC-LynTag-RFP). CP = cerebellar plate, DR = dorsal raphe, mlf = medial longitudinal fasciculus, OT = Optic Tectum, Va = valvula cerebelli. Inset shows location of slice, approximately at ZBBrowser coordinates [380, 513, 209].

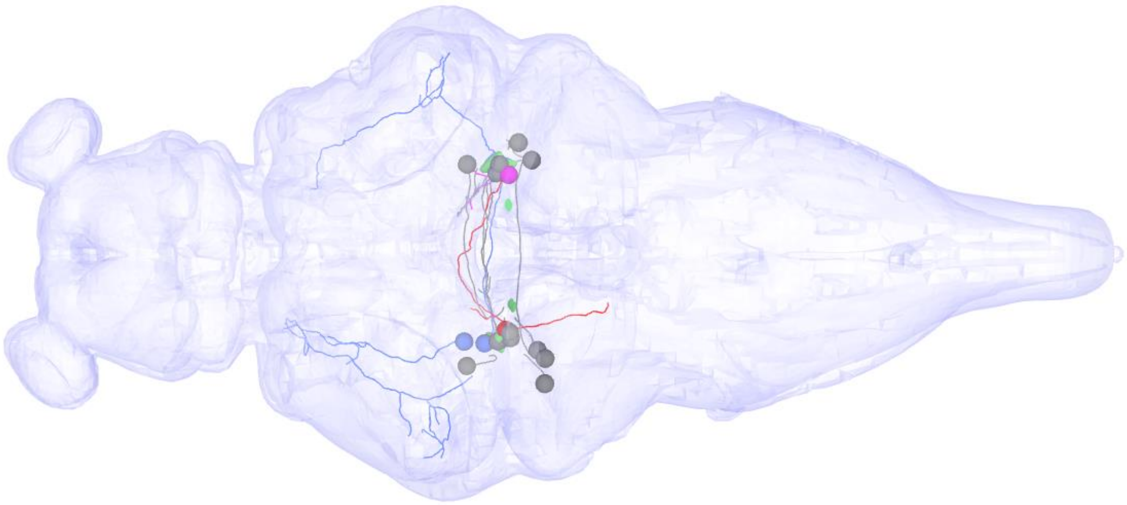

Fig. S7. vPPN projection patterns to contralateral vPPN and to hypothalamus

3D reconstructions of vPPNs from *y334-Gal4* in a model of the larval zebrafish brain (blue) with segmented RoL1 reticulospinal neurons (green). Representative examples of ipsilateral projecting (n = 7 neurons), contralateral projecting (n = 5), and hypothalamus projecting (n = 2) neurons can be seen in Figure 8B. For an interactive model of vPPN projections, please see the supplementary file, “Interactive 3D reconstruction of vPPNs”.

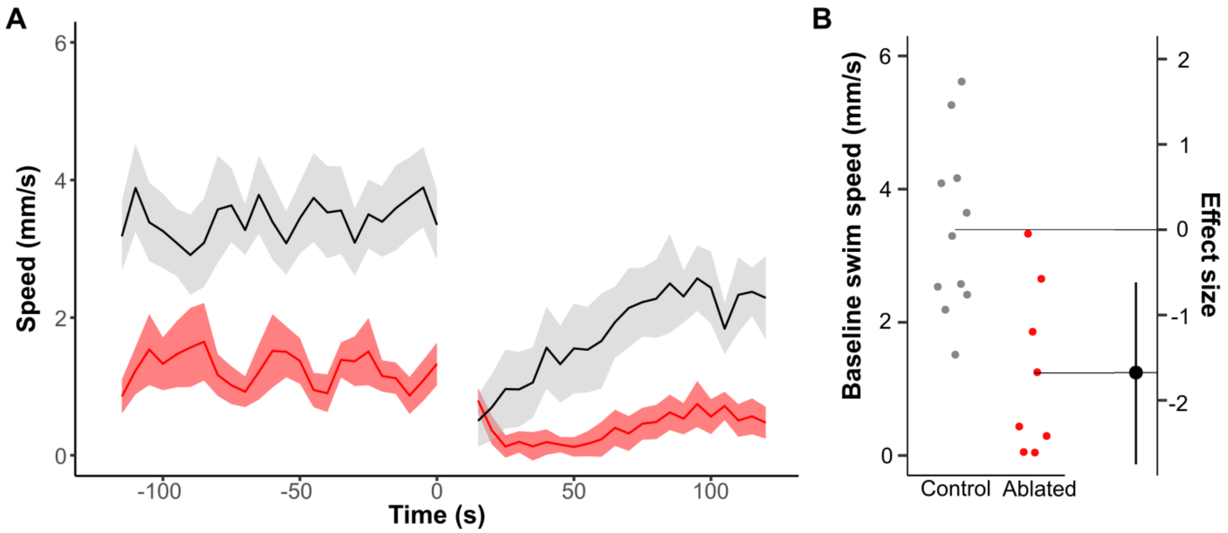

Fig. S8. Bilateral RoL1 neuron ablation suppresses baseline movement
(**A**) Time series trace of movement in RoL1 ablated fish (red, n = 8) and sham ablated controls (grey, n = 11 fish). (**B**) Mean baseline swim speed in RoL1 ablated neurons (red) compared to sham ablated controls (grey).

Other Supplementary Material for this manuscript includes the following

Supplementary Video 1.

Example of fish movement during vibratory stimulus used for behavioral experiments. Video was acquired at 500 frames/sec and is slowed by 10x.

Interactive 3D reconstruction of vPPNs

REFERENCES AND NOTES

1. W. R. Klemm, Behavioral arrest: In search of the neural control system. *Prog. Neurobiol.* **65**, 453–471 (2001).

2. T. Roseberry, A. Kreitzer, Neural circuitry for behavioural arrest. *Philos. Trans. R. Soc. Lond. B Biol. Sci.* **372**, 20160197 (2017).

3. W. T. Gibson, C. R. Gonzalez, C. Fernandez, L. Ramasamy, T. Tabachnik, R. R. Du, P. D. Felsen, M. R. Maire, P. Perona, D. J. Anderson, Behavioral responses to a repetitive visual threat stimulus express a persistent state of defensive arousal in Drosophila. *Curr. Biol.* **25**, 1401–1415 (2015).

4. F. Liang, X. R. Xiong, B. Zingg, X.-Y. Ji, L. I. Zhang, H. W. Tao, Sensory cortical control of a visually induced arrest behavior via corticotectal projections. *Neuron* **86**, 755–767 (2015).

5. R. Zacarias, S. Namiki, G. M. Card, M. L. Vasconcelos, M. A. Moita, Speed dependent descending control of freezing behavior in *Drosophila melanogaster*. *Nat. Commun.* **9**, 3697 (2018).

6. G. G. Gallup, Tonic immobility: The role of fear and predation. *Psychol. Rec.* **27**, 41–61 (1977).

7. K. Kozlowska, P. Walker, L. McLean, P. Carrive, Fear and the defense cascade: Clinical implications and management. *Harv. Rev. Psychiatry* **23**, 263–287 (2015).

8. M. A. Hagenaars, Tonic immobility and PTSD in a large community sample. *J. Exp. Psychopathol.* **7**, 246–260 (2016).

9. K. Roelofs, Freeze for action: Neurobiological mechanisms in animal and human freezing. *Philos. Trans. R. Soc. Lond. B Biol. Sci.* **372**, 20160206 (2017).

10. P. Tovote, M. S. Esposito, P. Botta, F. Chaudun, J. P. Fadok, M. Markovic, S. B. E. Wolff, C. Ramakrishnan, L. Fenno, K. Deisseroth, C. Herry, S. Arber, A. Lüthi, Midbrain circuits for defensive behaviour. *Nature* **534**, 206–212 (2016).

11. C. E. Vaaga, S. T. Brown, I. M. Raman, Cerebellar modulation of synaptic input to freezing-related neurons in the periaqueductal gray. *Elife* **9**, e54302 (2020).

12. R. K. Humphreys, G. D. Ruxton, A review of thanatosis (death feigning) as an anti-predator behaviour. *Behav. Ecol. Sociobiol.* **72**, 22 (2018).

13. E. Volchan, V. Rocha-Rego, A. F. Bastos, J. M. Oliveira, C. Franklin, S. Gleiser, W. Berger, G. G. L. Souza, L. Oliveira, I. A. David, F. S. Erthal, M. G. Pereira, I. Figueira, Immobility reactions under threat: A contribution to human defensive cascade and PTSD. *Neurosci. Biobehav. Rev.* **76**, 29–38 (2017).

14. F. T. Crawford, Induction and duration of tonic immobility. *Psychol. Rec.* **27**, 89–107 (1977).

15. W. R. Klemm, Identity of sensory and motor systems that are critical to the immobility reflex (“animal hypnosis”). *J. Neurosci. Res.* **2**, 57–69 (1976).

16. E. R. Duboué, E. Hong, K. C. Eldred, M. E. Halpern, Left habenular activity attenuates fear responses in larval Zebrafish. *Curr. Biol.* **27**, 2154–2162.e3 (2017).

17. S. Jesuthasan, S. Krishnan, R.-K. Cheng, A. Mathuru, Neural correlates of state transitions elicited by a chemosensory danger cue. *Prog. Neuropsychopharmacol. Biol. Psychiatry* **111**, 110110 (2021).

18. J. Cachat, A. Stewart, L. Grossman, S. Gaikwad, F. Kadri, K. M. Chung, N. Wu, K. Wong, S. Roy, C. Suciu, J. Goodspeed, M. Elegante, B. Bartels, S. Elkhayat, D. Tien, J. Tan, A. Denmark, T. Gilder, E. Kyzar, J. Dileo, K. Frank, K. Chang, E. Utterback, P. Hart, A. V. Kalueff, Measuring behavioral and endocrine responses to novelty stress in adult zebrafish. *Nat. Protoc.* **5**, 1786–1799 (2010).

19. M. Yoshida, in *Death-Feigning in Insects: Mechanism and Function of Tonic Immobility*, M. Sakai, Ed. (Springer Singapore, 2021), pp. 159–178.

20. T. Yokogawa, M. C. Hannan, H. A. Burgess, The dorsal raphe modulates sensory responsiveness during arousal in zebrafish. *J. Neurosci.* **32**, 15205–15215 (2012).

21. B. Jänicke, H. Coper, in *Advances in Psychology*, A.-M. Ferrandez, N. Teasdale, Eds. (North-Holland, 1996), vol. 114, pp. 201–233.

22. M. S. Fanselow, Neural organization of the defensive behavior system responsible for fear. *Psychon. Bull. Rev.* **1**, 429–438 (1994).

23. G. G. Gallup, D. R. Rager, in *Motor Activity and Movement Disorders: Research Issues and Applications*, P. R. Sanberg, K.-P. Ossenkopp, M. Kavaliers, Eds. (Humana Press, 1996), pp. 57–80.

24. M. W. Bagnall, D. L. McLean, Modular organization of axial microcircuits in zebrafish. *Science* **343**, 197–200 (2014).

25. K. M. Tabor, S. A. Bergeron, E. J. Horstick, D. C. Jordan, V. Aho, T. Porkka-Heiskanen, G. Haspel, H. A. Burgess, Direct activation of the Mauthner cell by electric field pulses drives ultrarapid escape responses. *J. Neurophysiol.* **112**, 834–844 (2014).

26. S. A. Bergeron, M. C. Hannan, H. Codore, K. Fero, G. H. Li, Z. Moak, T. Yokogawa, H. A. Burgess, Brain selective transgene expression in zebrafish using an NRSE derived motif. *Front. Neural Circuits.* **6**, 110 (2012).

27. G. D. Marquart, K. M. Tabor, M. Brown, J. L. Strykowski, G. K. Varshney, M. C. LaFave, T. Mueller, S. M. Burgess, S.-I. Higashijima, H. A. Burgess, A 3D searchable database of transgenic Zebrafish Gal4 and Cre lines for functional neuroanatomy studies. *Front. Neural Circuits.* **9**, 78 (2015).

28. K. M. Tabor, G. D. Marquart, C. Hurt, T. S. Smith, A. K. Geoca, A. A. Bhandiwad, A. Subedi, J. L. Sinclair, H. M. Rose, N. F. Polys, H. A. Burgess, Brain-wide cellular resolution imaging of Cre transgenic zebrafish lines for functional circuit-mapping. *Elife* **8**, e42687 (2019).

29. M. Takeuchi, S. Yamaguchi, Y. Sakakibara, T. Hayashi, K. Matsuda, Y. Hara, C. Tanegashima, T. Shimizu, S. Kuraku, M. Hibi, Gene expression profiling of granule cells and Purkinje cells in the zebrafish cerebellum. *J. Comp. Neurol.* **525**, 1558–1585 (2017).

30. R. Dohaku, M. Yamaguchi, N. Yamamoto, T. Shimizu, F. Osakada, M. Hibi, Tracing of afferent connections in the zebrafish cerebellum using recombinant rabies virus. *Front. Neural Circuits* **13**, 30 (2019).

31. S. M. Echteler, Organization of central auditory pathways in a teleost fish, *Cyprinus carpio*. *J. Comp. Physiol. A* **156**, 267–280 (1985).

32. P. M. Henriques, N. Rahman, S. E. Jackson, I. H. Bianco, Nucleus isthmi is required to sustain target pursuit during visually guided prey-catching. *Curr. Biol.* **29**, 1771–1786.e5 (2019).

33. M. Ekker, J. Wegner, M. A. Akimenko, M. Westerfield, Coordinate embryonic expression of three zebrafish engrailed genes. *Development* **116**, 1001–1010 (1992).

34. R. D. Palmiter, The parabrachial nucleus: CGRP neurons function as a general alarm. *Trends Neurosci.* **41**, 280–293 (2018).

35. Y.-K. Bae, S. Kani, T. Shimizu, K. Tanabe, H. Nojima, Y. Kimura, S.-I. Higashijima, M. Hibi, Anatomy of zebrafish cerebellum and screen for mutations affecting its development. *Dev. Biol.* **330**, 406–426 (2009).

36. J.-H. Son, M. D. Keefe, T. J. Stevenson, J. P. Barrios, S. Anjewierden, J. B. Newton, A. D. Douglass, J. L. Bonkowsky, Transgenic FingRs for live mapping of synaptic dynamics in genetically-defined neurons. *Sci. Rep.* **6**, 18734 (2016).

37. T. C. Harmon, D. L. McLean, I. M. Raman, Integration of swimming-related synaptic excitation and inhibition by olig2+ eurydendroid neurons in larval zebrafish cerebellum. *J. Neurosci.* **40**, 3063–3074 (2020).

38. M. Kunst, E. Laurell, N. Mokayes, A. Kramer, F. Kubo, A. M. Fernandes, D. Förster, M. Dal Maschio, H. Baier, A cellular-resolution atlas of the larval zebrafish brain. *Neuron* **103**, 21–38.e5 (2019).

39. W. Shen, J. S. Da Silva, H. He, H. T. Cline, Type A GABA-receptor-dependent synaptic transmission sculpts dendritic arbor structure in Xenopus tadpoles in vivo. *J. Neurosci.* **29**, 5032–5043 (2009).

40. O. Randlett, C. L. Wee, E. A. Naumann, O. Nnaemeka, D. Schoppik, J. E. Fitzgerald, R. Portugues, A. M. B. Lacoste, C. Riegler, F. Engert, A. F. Schier, Whole-brain activity mapping onto a zebrafish brain atlas. *Nat. Methods* **12**, 1039–1046 (2015).

41. Y. Dai, K. Iwata, T. Fukuoka, E. Kondo, A. Tokunaga, H. Yamanaka, T. Tachibana, Y. Liu, K. Noguchi, Phosphorylation of extracellular signal-regulated kinase in primary afferent neurons by noxious stimuli and its involvement in peripheral sensitization. *J. Neurosci.* **22**, 7737–7745 (2002).

42. K. S. Liu, J. R. Fetcho, Laser ablations reveal functional relationships of segmental hindbrain neurons in zebrafish. *Neuron* **23**, 325–335 (1999).

43. D. Förster, M. Dal Maschio, E. Laurell, H. Baier, An optogenetic toolbox for unbiased discovery of functionally connected cells in neural circuits. *Nat. Commun.* **8**, 116 (2017).

44. P. Antinucci, A. Dumitrescu, C. Deleuze, H. J. Morley, K. Leung, T. Hagley, F. Kubo, H. Baier, I. H. Bianco, C. Wyart, A calibrated optogenetic toolbox of stable zebrafish opsin lines. *Elife* **9**, e54937 (2020).

45. K. M. Tabor, T. S. Smith, M. Brown, S. A. Bergeron, K. L. Briggman, H. A. Burgess, Presynaptic inhibition selectively gates auditory transmission to the brainstem startle circuit. *Curr. Biol.* **28**, 2527–2535.e8 (2018).

46. M. Lovett-Barron, R. Chen, S. Bradbury, A. S. Andalman, M. Wagle, S. Guo, K. Deisseroth, Multiple convergent hypothalamus-brainstem circuits drive defensive behavior. *Nat. Neurosci.* **23**, 959–967 (2020).

47. M. B. Orger, A. R. Kampff, K. E. Severi, J. H. Bollmann, F. Engert, Control of visually guided behavior by distinct populations of spinal projection neurons. *Nat. Neurosci.* **11**, 327–333 (2008).

48. P. H. Edson, G. G. Gallup, Tonic immobility as a fear response in lizards Anolis carolinensis. *Psychon. Sci.* **26**, 27–28 (1972).

49. G. Esposito, S. Yoshida, R. Ohnishi, Y. Tsuneoka, M. D. C. Rostagno, S. Yokota, S. Okabe, K. Kamiya, M. Hoshino, M. Shimizu, P. Venuti, T. Kikusui, T. Kato, K. O. Kuroda, Infant calming responses during maternal carrying in humans and mice. *Curr. Biol.* **23**, 739–745 (2013).

50. A. L. Person, I. M. Raman, Purkinje neuron synchrony elicits time-locked spiking in the cerebellar nuclei. *Nature* **481**, 502–505 (2011).

51. C. R. Monassi, C. R. Leite-Panissi, L. Menescal-de-Oliveira, Ventrolateral periaqueductal gray matter and the control of tonic immobility. *Brain Res. Bull.* **50**, 201–208 (1999).

52. M. Hashimoto, A. Yamanaka, S. Kato, M. Tanifuji, K. Kobayashi, H. Yaginuma, Anatomical evidence for a direct projection from purkinje cells in the mouse cerebellar vermis to medial parabrachial nucleus. *Front. Neural Circuits.* **12**, 6 (2018).

53. S. L. Alderman, N. J. Bernier, Ontogeny of the corticotropin-releasing factor system in zebrafish. *Gen. Comp. Endocrinol.* **164**, 61–69 (2009).

54. R. L. Spinieli, C. R. A. Leite-Panissi, Similar effect of CRF1 and CRF2 receptor in the basolateral or central nuclei of the amygdala on tonic immobility behavior. *Brain Res. Bull.* **137**, 187–196 (2018).

55. N. J. Bernier, S. L. Alderman, E. N. Bristow, Heads or tails? Stressor-specific expression of corticotropin-releasing factor and urotensin I in the preoptic area and caudal neurosecretory system of rainbow trout. *J. Endocrinol.* **196**, 637–648 (2008).

56. S.-Y. Lv, Y.-C. Zhou, X.-M. Zhang, W.-D. Chen, Y.-D. Wang, Emerging roles of NPQ/Spexin in physiology and pathology. *Front. Pharmacol.* **10**, 457 (2019).

57. R. J. Valentino, C. Rudoy, A. Saunders, X. B. Liu, E. J. Van Bockstaele, Corticotropin-releasing factor is preferentially colocalized with excitatory rather than inhibitory amino acids in axon terminals in the peri-locus coeruleus region. *Neuroscience* **106**, 375–384 (2001).

58. L. Menescal-de-Oliveira, A. Hoffmann, The parabrachial region as a possible region modulating simultaneously pain and tonic immobility. *Behav. Brain Res.* **56**, 127–132 (1993).

59. A. S. Andalman, V. M. Burns, M. Lovett-Barron, M. Broxton, B. Poole, S. J. Yang, L. Grosenick, T. N. Lerner, R. Chen, T. Benster, P. Mourrain, M. Levoy, K. Rajan, K. Deisseroth, Neuronal dynamics regulating brain and behavioral state transitions. *Cell* **177**, 970–985.e20 (2019).

60. Y. Yang, Y. Cui, K. Sang, Y. Dong, Z. Ni, S. Ma, H. Hu, Ketamine blocks bursting in the lateral habenula to rapidly relieve depression. *Nature* **554**, 317–322 (2018).

61. W. F. Supple Jr, J. Cranney, R. N. Leaton, Effects of lesions of the cerebellar vermis on VMH lesion-induced hyperdefensiveness, spontaneous mouse killing, and freezing in rats. *Physiol. Behav.* **42**, 145–153 (1988).

62. B. A. Silva, C. T. Gross, J. Gräff, The neural circuits of innate fear: Detection, integration, action, and memorization. *Learn. Mem.* **23**, 544–555 (2016).

63. S. Koutsikou, J. J. Crook, E. V. Earl, J. L. Leith, T. C. Watson, B. M. Lumb, R. Apps, Neural substrates underlying fear-evoked freezing: The periaqueductal grey-cerebellar link. *J. Physiol.* **592**, 2197–2213 (2014).

64. A. J. Bowen, J. Y. Chen, Y. W. Huang, N. A. Baertsch, S. Park, R. D. Palmiter, Dissociable control of unconditioned responses and associative fear learning by parabrachial CGRP neurons. *Elife* **9**, e59799 (2020).

65. D. J. Anderson, R. Adolphs, A framework for studying emotions across species. *Cell* **157**, 187–200 (2014).

66. K. Fero, S. A. Bergeron, E. J. Horstick, H. Codore, G. H. Li, F. Ono, J. J. Dowling, H. A. Burgess, Impaired embryonic motility in dusp27 mutants reveals a developmental defect in myofibril structure. *Dis. Model. Mech.* **7**, 289–298 (2014).

67. H. A. Burgess, M. Granato, Sensorimotor gating in larval zebrafish. *J. Neurosci.* **27**, 4984–4994 (2007).

68. H. A. Burgess, M. Granato, Modulation of locomotor activity in larval zebrafish during light adaptation. *J. Exp. Biol.* **210**, 2526–2539 (2007).

69. J. A. Harris, A. G. Cheng, L. L. Cunningham, G. MacDonald, D. W. Raible, E. W. Rubel, Neomycin-induced hair cell death and rapid regeneration in the lateral line of zebrafish (*Danio rerio*). *J. Assoc. Res. Otolaryngol.* **4**, 219–234 (2003).

70. C. M. Hempel, K. Sugino, S. B. Nelson, A manual method for the purification of fluorescently labeled neurons from the mammalian brain. *Nat. Protoc.* **2**, 2924–2929 (2007).

71. S. Anders, W. Huber, Differential expression analysis for sequence count data. *Genome Biol.* **11**, R106 (2010).

72. G. D. Marquart, K. M. Tabor, E. J. Horstick, M. Brown, A. K. Geoca, N. F. Polys, D. D. Nogare, H. A. Burgess, High-precision registration between zebrafish brain atlases using symmetric diffeomorphic normalization. *Gigascience* **6**, 1–15 (2017).

73. B. B. Avants, N. J. Tustison, G. Song, P. A. Cook, A. Klein, J. C. Gee, A reproducible evaluation of ANTs similarity metric performance in brain image registration. *Neuroimage* **54**, 2033–2044 (2011).

74. H. M. T. Choi, M. Schwarzkopf, M. E. Fornace, A. Acharya, G. Artavanis, J. Stegmaier, A. Cunha, N. A. Pierce, Third-generation in situ hybridization chain reaction: Multiplexed, quantitative, sensitive, versatile, robust. *Development* **145**, dev16573 (2018).

75. J. Ho, T. Tumkaya, S. Aryal, H. Choi, A. Claridge-Chang, Moving beyond P values: Data analysis with estimation graphics. *Nat. Methods* **16**, 565–566 (2019).

76. M. Takeuchi, K. Matsuda, S. Yamaguchi, K. Asakawa, N. Miyasaka, P. Lal, Y. Yoshihara, A. Koga, K. Kawakami, T. Shimizu, M. Hibi, Establishment of Gal4 transgenic zebrafish lines for analysis of development of cerebellar neural circuitry. *Dev. Biol.* **397**, 1–17 (2015).

77. A. M. Fernandes, K. Fero, A. B. Arrenberg, S. A. Bergeron, W. Driever, H. A. Burgess, Deep brain photoreceptors control light-seeking behavior in zebrafish larvae. *Curr. Biol.* **22**, 2042–2047 (2012).

78. C. Satou, Y. Kimura, H. Hirata, M. L. Suster, K. Kawakami, S.-I. Higashijima, Transgenic tools to characterize neuronal properties of discrete populations of zebrafish neurons. *Development* **140**, 3927–3931 (2013).

79. W. Mo, T. Nicolson, Both pre- and postsynaptic activity of Nsf prevents degeneration of hair-cell synapses. *PLOS ONE* **6**, e27146 (2011).
