# Supplementary material for: A cerebellar-prepontine circuit for tonic immobility triggered by an inescapable threat
Source: Sci Adv. 2022 Sep 28;8(39):eabo0549. doi: 10.1126/sciadv.abo0549 (PMC9519051; doi:10.1126/sciadv.abo0549)
Supplement: Supplementary file 3 — Interactive 3D reconstruction of vPPNs [file sciadv.abo0549_interactive_3d_reconstruction_of_vppns.zip › sciadv.abo0549_interactive_3d_reconstruction_of_vppns.html]

ZBB Projection


    

   


    

   


    

   


    

   


    

   


    

   


    

   


    

   


    

   


    

   


    

   


    

   


    

   


    

   


    

   


    

   
